# Exploring the biological roles of Dothideomycetes ABC proteins: Leads from their phylogenetic relationships with functionally-characterized Ascomycetes homologs

**DOI:** 10.1371/journal.pone.0197447

**Published:** 2018-08-02

**Authors:** Gaurav Dube, Narendra Kadoo, Ramya Prashant

**Affiliations:** 1 Biochemical Sciences Division, CSIR-National Chemical Laboratory, Pune, India; 2 Academy of Scientific and Innovative Research (AcSIR), New Delhi, India; 3 MIT School of Bioengineering Sciences & Research, MIT-Art, Design and Technology University, Pune, India; Universidade Nova de Lisboa Instituto de Tecnologia Quimica e Biologica, PORTUGAL

## Abstract

**Background:**

The ATP-binding cassette (ABC) superfamily is one of the largest, ubiquitous and diverse protein families in nature. Categorized into nine subfamilies, its members are important to most organisms including fungi, where they play varied roles in fundamental cellular processes, plant pathogenesis or fungicide tolerance. However, these proteins are not yet well-understood in the class Dothideomycetes, which includes several phytopathogens that infect a wide range of food crops including wheat, barley and maize and cause major economic losses.

**Results:**

We analyzed the genomes of 14 Dothideomycetes fungi (Test set) and seven well-known Ascomycetes fungi (Model set- that possessed gene expression/ functional analysis data about the ABC genes) and predicted 578 and 338 ABC proteins from each set respectively. These proteins were classified into subfamilies A to I, which revealed the distribution of the subfamily members across the Dothideomycetes and Ascomycetes genomes. Phylogenetic analysis of Dothideomycetes ABC proteins indicated evolutionary relationships among the subfamilies within this class. Further, phylogenetic relationships among the ABC proteins from the Model and the Test fungi within each subfamily were analyzed, which aided in classifying these proteins into subgroups. We compiled and curated functional and gene expression information from the previous literature for 118 ABC genes and mapped them on the phylogenetic trees, which suggested possible roles in pathogenesis and/or fungicide tolerance for the newly identified Dothideomycetes ABC proteins.

**Conclusions:**

The present analysis is one of the firsts to extensively analyze ABC proteins from Dothideomycetes fungi. Their phylogenetic analysis and annotating the clades with functional information indicated a subset of Dothideomycetes ABC genes that could be considered for experimental validation for their roles in plant pathogenesis and/or fungicide tolerance.

## Introduction

Dothideomycetes forms the largest and one of the most diverse classes of the phylum Ascomycetes [1]. The fungal species belonging to this class survive in diverse ecologies and have capabilities to adapt to various environments and habitats [2]. Several of these fungi can infect a wide array of plants from terrestrial to aquatic habitats through different pathogenic lifestyles like biotrophy, hemibiotrophy or necrotrophy [3–5]. Several Dothideomycetes fungal pathogens prominently of the orders Capnodiales, Pleosporales, Botryosphaeriales and Venturiales have evolved high level of host-specificity [6], resulting in the emergence of various diseases like rot, blight, blotch, mold, spot and scab in plants. The pathogenic invasion of this fungal class is so extensive that its members can infect every major agricultural crop including wheat and rice [3] affecting their growth, development, yield, quality and ultimately hampering food, fodder, fuel and fiber products.

The Dothideomycetes phytopathogens deal not only with plant defense molecules during their establishment in the host but also with a plethora of fungicidal chemicals that modern agriculture employs for crop protection. To achieve sustenance (nutrient acquisition, growth, development, reproduction) and survival (virulence/ defense; invasion/ co-existence), these fungi dynamically modulate their interactions with the surrounding biotic and abiotic components. Efficient cellular transportation that carries out efflux and influx of natural or artificial compounds is one of the important systems required for interactions with the environment. One of such transport systems is mediated by the ATP-binding cassette (ABC) superfamily proteins.

Most of the ABC superfamily proteins are involved in transport of an extensive range of substrates across the cellular membranes using ATP-derived energy [7]. A subset of the members of this superfamily also play crucial roles in fundamental cellular processes such as ribosome biogenesis, translation, DNA repair, regulation of gene expression and receptor signaling ([8] and references therein). Its members are reported from archaea, bacteria and eukaryotes and they constitute one of the largest gene families known to us. The ABC proteins are classified into eight major subfamilies from A to H. An additional subfamily I has also been proposed that includes prokaryotic-type transporters encoded by eukaryotic genomes [9,10]. Subfamily H genes occur in prokaryotes, insects and fishes, but its members have not yet been reported in plants and fungi [9].

**Fig 1:**
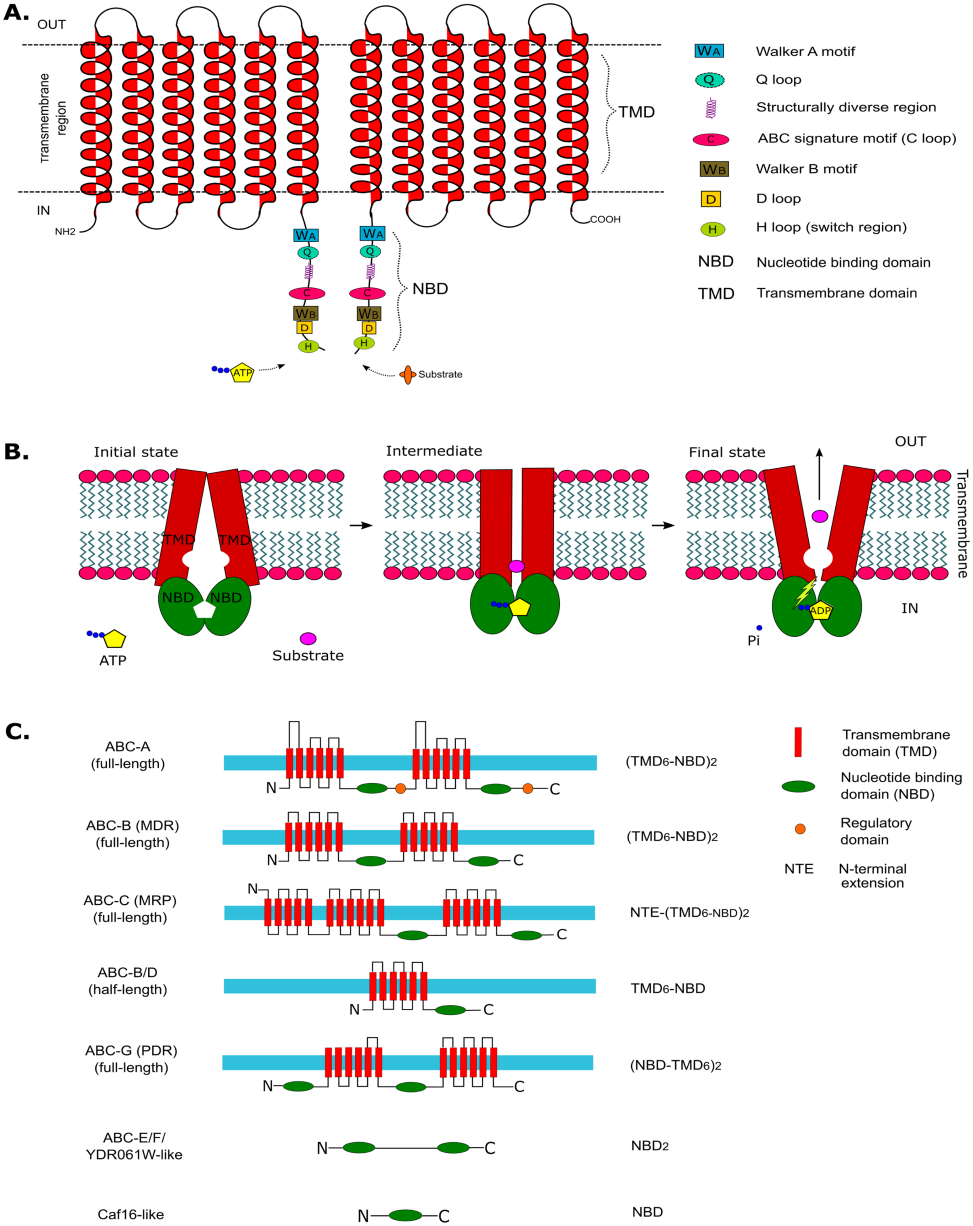
Schematic representation of: A) Canonical structure of ABC proteins. Walker-A, ABC-signature and Walker-B are characteristic motifs found in all ABC ATPases. B) The typical mechanism of an ABC exporter. C) Predicted topology and domain organization in different subfamilies of fungal ABC proteins.

The canonical structure of an ABC protein (Fig 1A) consists of nucleotide binding domain (NBD) and transmembrane domain (TMD) organized in conserved modular fashion [7]. The NBD consists of three characteristic motifs: Walker-A (GxxGxGKS/T, where x is any amino acid), Walker-B (####D, where # is a hydrophobic amino acid) and the LSGGQ (ABC-signature/ C-loop/ linker peptide). In addition to these characteristic motifs, other conserved motifs like Q-loop, D-loop and H-loop with specific functions are also present as parts of the NBD-NBD interface [11] (Fig 1A). The ABC proteins depend on ATP energy to transport their substrates (Fig 1B). They differ in their domain organization and topology in terms of the number of NBD, TMD and their order (Fig 1C). ABC proteins possessing two sets of NBD and TMD (members of subfamilies A, B, C and G) are known as full transporters (FT); while the half transporters (HT; subfamilies B, D and G) possess a single set. The ABC E, F and I subfamily members are soluble proteins consisting of only one or two NBD and lack TMD [7,11].

In prokaryotes, majority of the ABC proteins are involved in import of essential nutrients. For example, the *Escherichia coli* BtuCD is known to mediate the import of vitamin B12 [12]; while ModBC of *Archaeoglobus fulgidus* is involved in molybdenum influx [13]. The eukaryotic ABC proteins transport diverse substrates including toxins, metal ions, fatty acids and secondary metabolites across membranes [14]. For instance, GintABC1 of the fungus *Glomus intraradices* is involved in Cu and Cd efflux [15] and *Candida albicans* Cdr1p and Cdr2p export fluconazole and carry out cellular detoxification [16]. Notably, several studies have demonstrated the involvement of fungal ABC genes in virulence and fungicide tolerance. For example, *MgAtr4* from the fungal pathogen *Mycosphaerella graminicola* acts as a virulence factor during wheat infection [17], while *Fusarium graminearum FgABC3* is responsible for virulence as well as azole tolerance [18]. Similarly, *ABC4* of *Magnaporthe oryzae* is necessary for appressoria formation and virulence as well as confers tolerance to antifungal compounds and phytoalexins [19]. These examples highlight the importance of ABC proteins in fungal survival and virulence and underline the need for studying them in view of designing better crop protection strategies.

Despite their importance in virulence, defense and resistance, ABC proteins have been poorly studied with very few genome-wide efforts to understand their roles in fungi. Particularly in Dothideomycetes, extensive work pertaining to ABC protein repertoire and phylogenetic relationships of the ABC subfamilies has not yet been performed. Kovalchuk and Driessen [10] described phylogenetic relationships among fungal ABC proteins from various classes including Dothideomycetes; however, they analyzed only two species from this class namely, *Stagonospora nodorum* and *Pyrenophora tritici-repentis*. Lamping *et al*. [20] performed phylogenetic analysis of pleiotropic drug resistance (PDR) transporters (subfamily G members) alone and included only six Dothideomycetes species in their analysis, *viz*. *Leptosphaeria maculans*, *M*. *graminicola*, *S*. *nodorum*, *P*. *tritici-repentis*, *Venturia inaequalis* and *Alternaria brassicicola*. Thus, considering their importance in plant-fungal interactions and fungicide tolerance, phylogenomic study of the ABC superfamily in Dothideomycetes, which harbors several important plant pathogens, would be valuable for increased understanding of these proteins.

**Fig 2:**
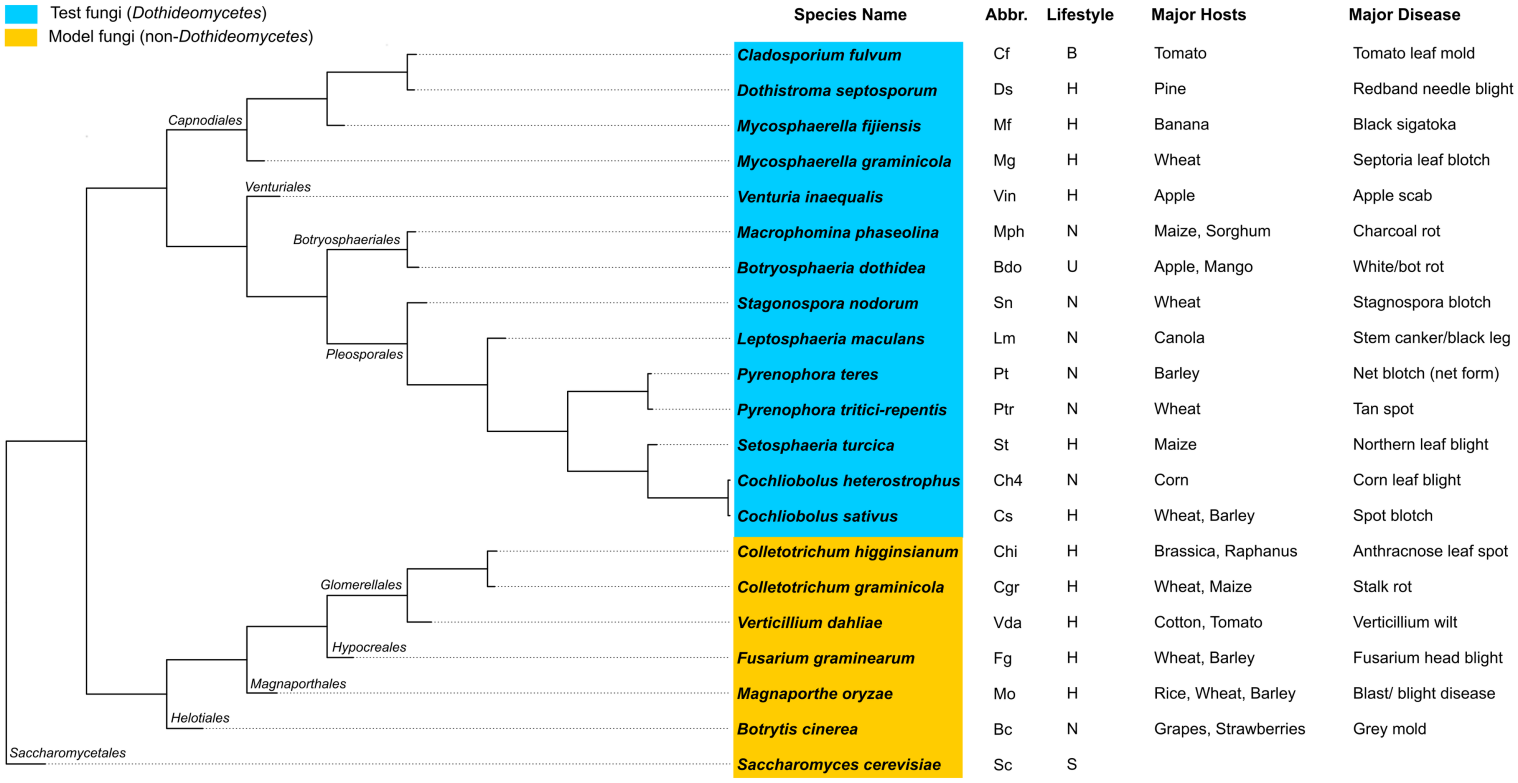
List of the analyzed fungi, their phylogenetic relationships, major hosts and related diseases. Orders of the species are specified in italics near the respective branches. The abbreviations (Abbr.) correspond to the names of the species and are used to refer to them in short-form in gene names throughout the text. Lifestyles: B-Biotrophic, H- Hemibiotrophic, N-Necrotrophic, U-undefined lifestyle, S-Saprophytic.

The recent rapid developments in fungal genomics provided us an opportunity for detailed investigation of the ABC superfamily in Dothideomycetes at the sequence level and motivated us to explore their ABC protein repertoire, the evolutionary relationships among them as well as their possible roles in pathogenicity and fungicide tolerance. Considering the genomic resources available, we selected 21 fungal species and categorized them into ‘Model’ and ‘Test’ (Fig 2; S1 Table). The ‘Model’ species included (i) *Saccharomyces cerevisiae* that harbored well-characterized ABC proteins, which served as the reference for predicting ABC proteins from the fungal genomes, and (ii) six Ascomycetes fungal phytopathogens from the classes Sordariomycetes and Leotiomycetes, which are phylogenetically close to the Dothideomycetes lineage [21], and the participation of a subset of their ABC genes in plant-pathogen interactions and/or fungicide tolerance had previously been demonstrated either by mutant or knock-out experiments or surveyed by gene expression studies. Alternatively, the ‘Test’ species included well-known phytopathogens from Dothideomycetes, but poorly studied from the ABC superfamily context. The rationale behind this categorization was, close phylogenetic clustering of ABC proteins from the Test and the Model fungi would allow extending the annotations of the ABC proteins from the Model fungi with functional/gene expression information, to the Test Dothideomycetes ABC proteins; proposing their putative functions, which could later be validated experimentally.

In the present study, we identified ABC proteins from the selected fungal genomes, classified them into their respective subfamilies and subjected them to phylogenetic analysis. The ABC protein annotations were obtained through data mining, and curating and re-analyzing the publicly available data. We surveyed the outcomes of several gene expression and functional studies performed previously on plant-pathogen interactions and fungicide tolerance, which were available in the public domain for the selected fungi in the form of RNAseq, microarray, qRT-PCR (quantitative reverse transcriptase PCR), SAGE (serial analysis of gene expression), knockout and mutant analyses. Mapping these annotations onto the phylogenetic trees provided a platform for associating the pathogenicity and/or fungicide tolerance-related functions of well-characterized ABC genes to their phylogenetically proximal Dothideomycetes counterparts and provided with a subset of Dothideomycetes ABC genes that could be directed for functional characterization experiments with better prospects.

## Materials and methods

### Fungal genomes

The Model fungi set included *Botrytis cinerea* (Bc), *Colletotrichum graminicola* (Cgr) (syn. *Glomerella graminicola*), *Colletotrichum higginsianum* (Chi), *Verticillium dahliae* (Vda), *Fusarium graminearum* (Fg) and *Magnaporthe oryzae* (Mo) (syn. *Magnaporthe grisea*). Additionally, *S*. *cerevisiae* (Sc) from the class Saccharomycetes was used as a reference for predicting ABC proteins from the genomes of all the considered fungi in this study. The ‘Test’ fungi included the Dothideomycetes *Stagonospora nodorum* (Sn) (syn. *Parastagonospora nodorum*/ *Phaeosphaeria nodorum*), *Setosphaeria turcica* (St), *Leptosphaeria maculans* (Lm), *Dothistroma septosporum* (Ds) (syn. *Mycosphaerella pini*), *Cladosporium fulvum* (Cf) (syn. *Passalora fulva*), *Mycosphaerella graminicola* (Mg) (syn. *Zymoseptoria tritici*), *Mycosphaerella fijiensis* (Mf) (syn. *Pseudocercospora fijiensis*), *Cochliobolus heterostrophus* (Ch4), *Cochliobolus sativus* (Cs), *Pyrenophora teres* (Pt), *Pyrenophora tritici-repentis* (Ptr), *Botryosphaeria dothidea* (Bdo), *Venturia inaequalis* (Vin) and *Macrophomina phaseolina* (Mph). The proteomic datasets of 14 Dothideomycetes, five Sordariomycetes and one Leotiomycetes fungi were retrieved from JGI-Mycocosm [22] (the genome information, isolate details, references and download links are provided in S1 Table).

### Identification of ABC proteins

The proteome datasets of all the selected fungi were subjected to InterProScan v5.8–49.0 [23] for identifying the ABC protein specific domains, i.e. NBD and TMD. Only those sequences, for which at least one NBD (Pfam ID Pf00005) was identified by InterProScan, were used as query against the ABC protein reference dataset in the BLASTp search analysis. The ABC protein reference dataset contained 29 experimentally validated members from *S*. *cerevisiae* downloaded from Swiss-Prot [24], of which 24 were functionally characterized previously (S2 Table). Additionally, three ABC subfamily-A (ABC-A) sequences from *Aspergillus nidulans* and *Dictyostelium discoideum* from UniProtKB [24] were included, because *S*. *cerevisiae* does not possess ABC-A genes. BLASTp analysis was performed with an e-value cutoff of ≤10^−6^. The best hits were classified into the respective major ABC protein subfamilies (A-I). Additionally, for these hits, the subfamily-specific domains i.e. PF12698 (ABC-A); PF00664 (ABC-B or C); PF06472 (ABC-D); PF00037, PF04068 (ABC-E); PF12848, PF00385 (ABC-F) and PF06422, PF01061, PF14510 (ABC-G) identified through InterProScan were noted, appended to the BLAST results and were additionally used to aid the classification of query sequences into their respective ABC protein subfamilies. These domains, along with their topologies, were used as pointers for confirming the classification. According to the number of NBDs possessed by each ABC protein, it was further classified into half transporter (HT, one NBD) or full transporter (FT, two NBDs).

### Phylogenetic analysis

The phylogenetic relationships among the fungi were assessed using the tree (Fig 2) generated by OrthoFinder v1.0.6 tool [25]. The whole protein sequence datasets of the 21 analyzed species (downloaded via the respective genome portal links provided in S1 Table, column N) were compiled and used as the input. First, the tool finds protein orthologs and orthogroups from the input dataset. Then, it uses median distances for the most closely related genes i.e. best orthologs between those species in the orthogroups to derive dendroblast distances for the species tree. Lastly, OrthoFinder implements STRIDE (http://biorxiv.org/content/early/2017/05/19/140020) for rooting of the species and generates a Newick file as a result (provided in S1–S10 Files).

**Fig 3:**
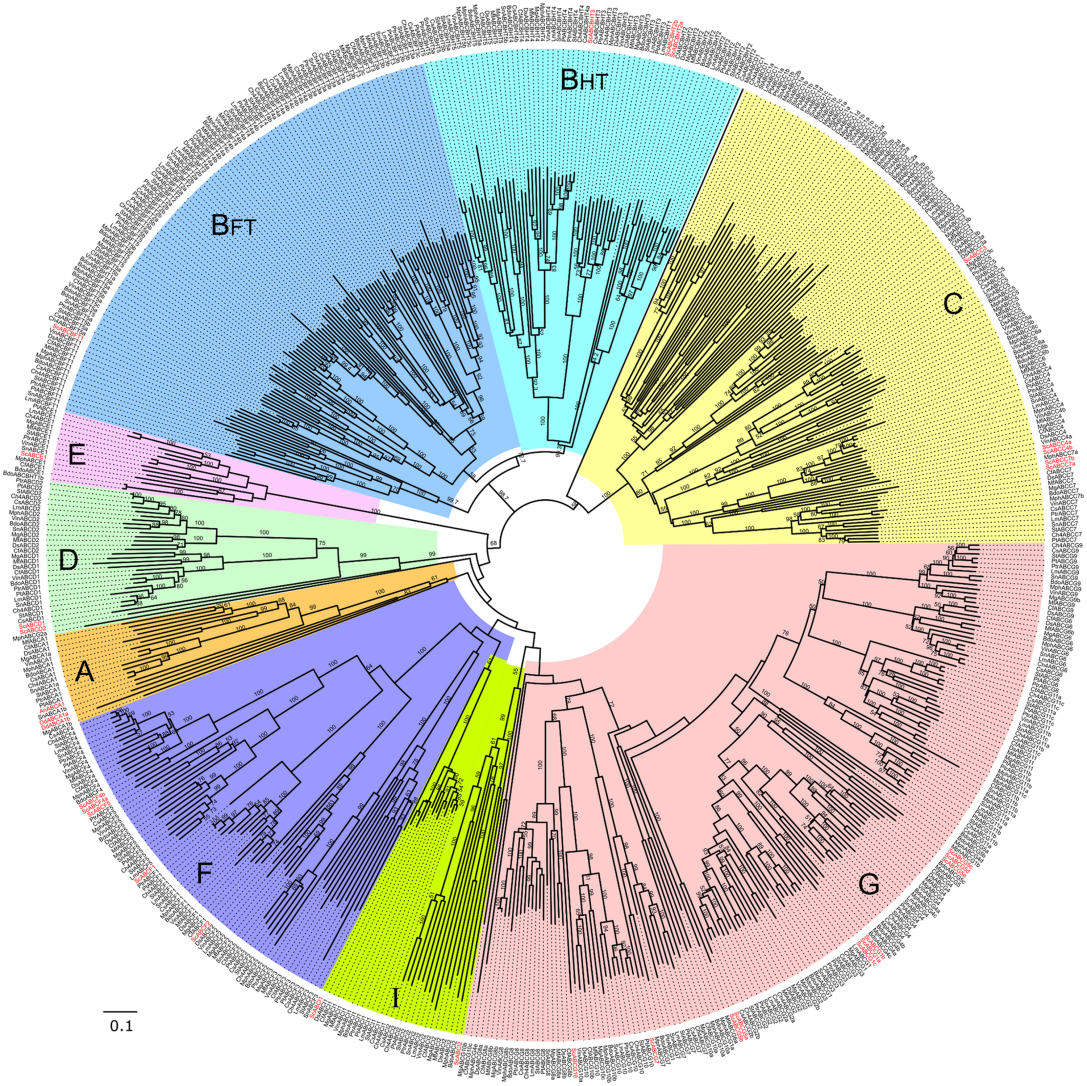
Phylogenetic tree (‘ABC-Dot’ tree) representing evolutionary relationships among ABC protein subfamilies of the 14 Dothideomycetes fungi. The black letters on the colored clades indicate respective ABC protein subfamilies. The numbers near the branches correspond to the bootstrap value for that branch (only bootstrap values ≥50 are shown). Node labels indicate ABC protein IDs represented in ‘<species abbreviation>ABC<subfamily><group number>‘ format (S8 Table) [10]. Node labels of reference ABC proteins from *S*. *cerevisiae*, *A*. *nidulans* and *D*. *discoideum* are highlighted in red.

**Fig 4:**
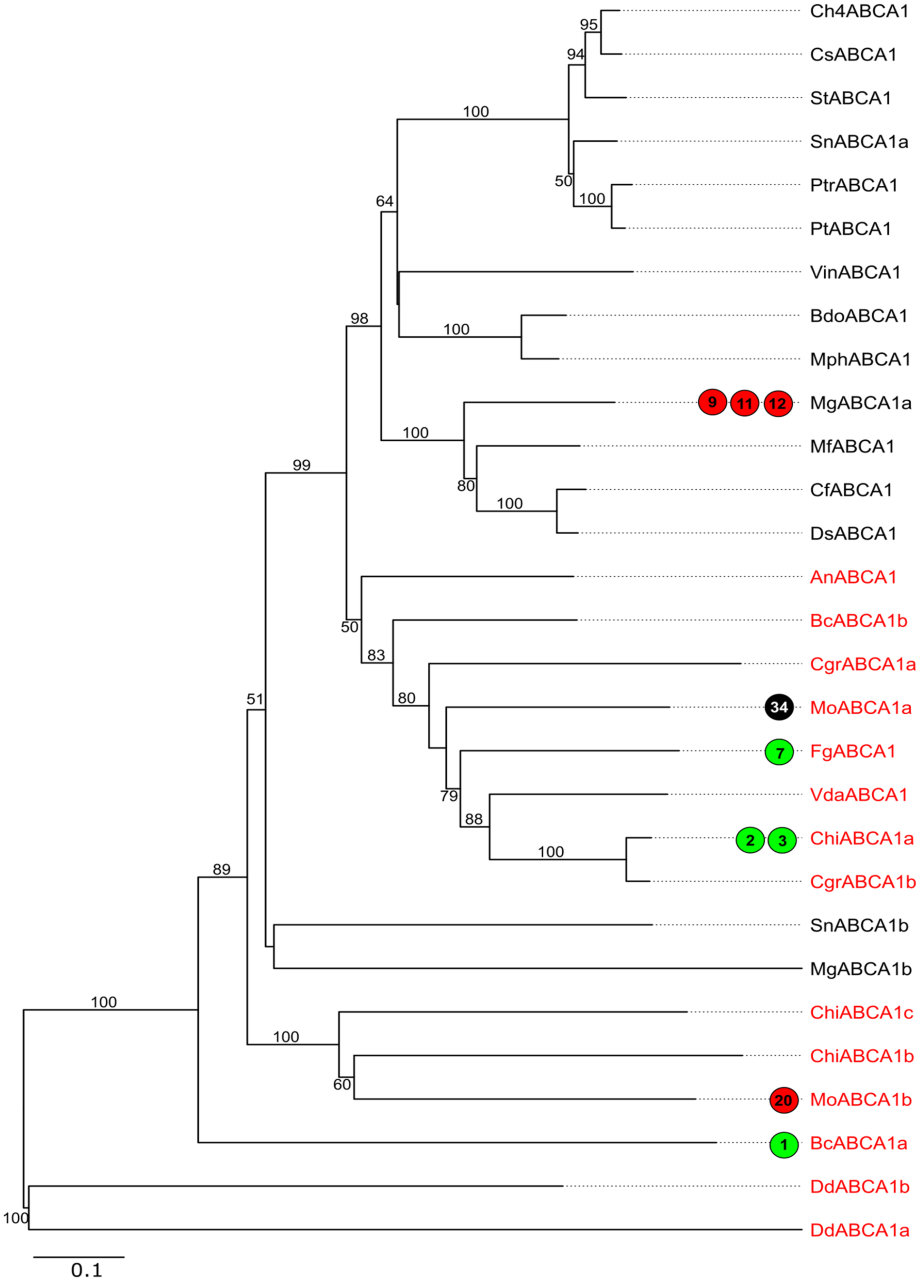
Phylogenetic tree of fungal ABC-A proteins. In this NJ tree, the numbers near the branches correspond to the bootstrap values (only bootstrap values ≥50 are shown). Node labels indicate ABC protein IDs (S8 Table). Proteins from Model fungi are highlighted in red labels and the Dothideomycetes ABC proteins are in black. The circles with green and red colors indicate that the corresponding ABC gene is up-regulated and down-regulated respectively in the experiment denoted by the number in the circle, whose information could be found in S5 Table. The black circles indicate that the corresponding ABC genes have undergone functional analyses denoted by the numbers in the circles, whose information is tabulated in S3 and S4 Tables.

**Fig 5:**
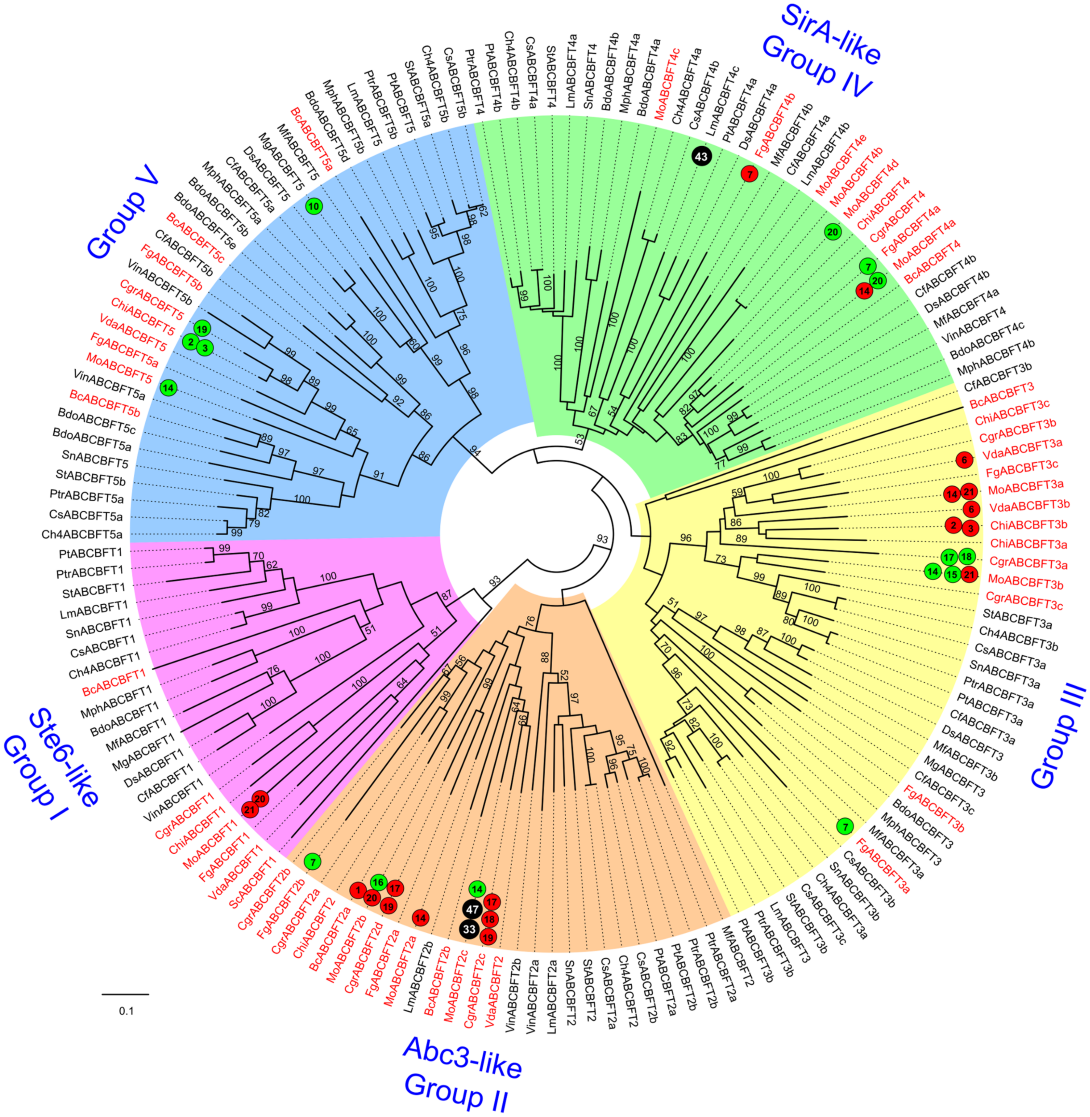
Neighbor Joining phylogenetic tree of fungal ABC-B full-transporter proteins. The tree was annotated as described for Fig 4. The names of the clades in the tree, if given, are after the well-characterized ABC genes present in the respective clades. Each of the colored clade could be considered a functional group comprising homologous members of the well-characterized ABC proteins with possibly similar functions.

**Fig 6:**
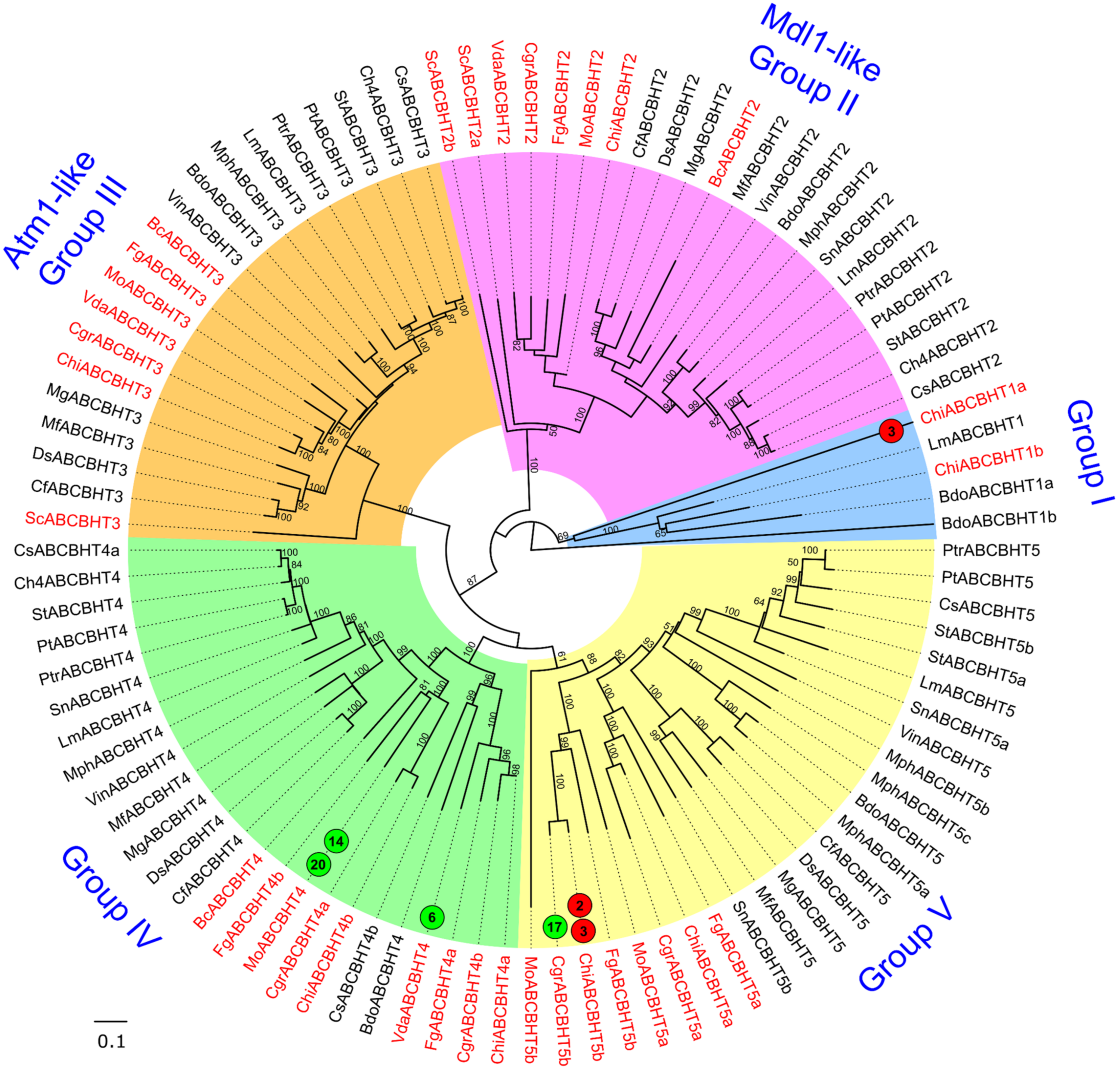
Phylogenetic tree of fungal ABC-B half-transporter proteins. The tree was generated and annotated as described in Figs 4 and 5.

**Fig 7:**
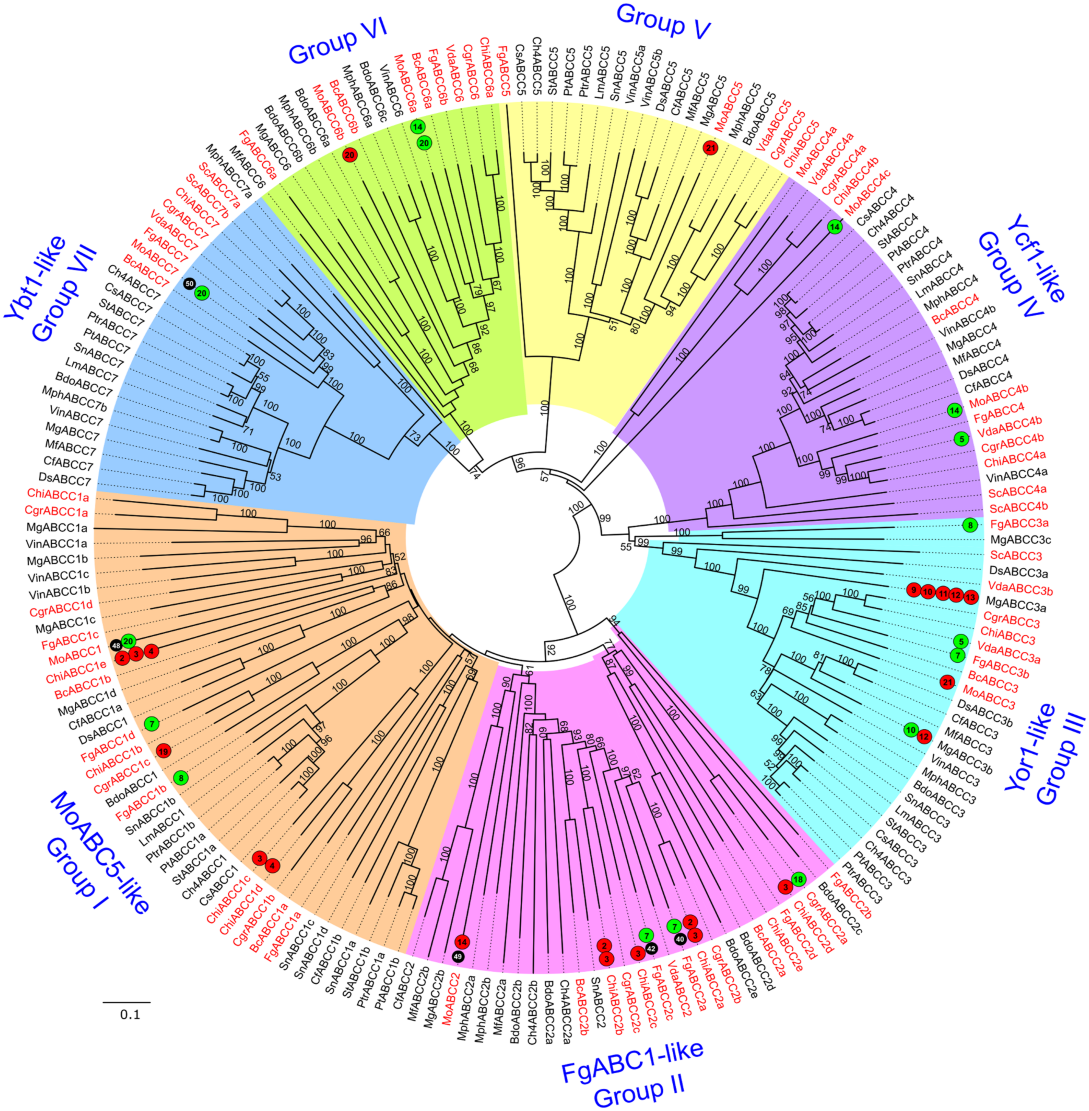
Phylogenetic tree of fungal ABC-C proteins. The tree was generated and annotated as described in case of Figs 4 and 5.

**Fig 8:**
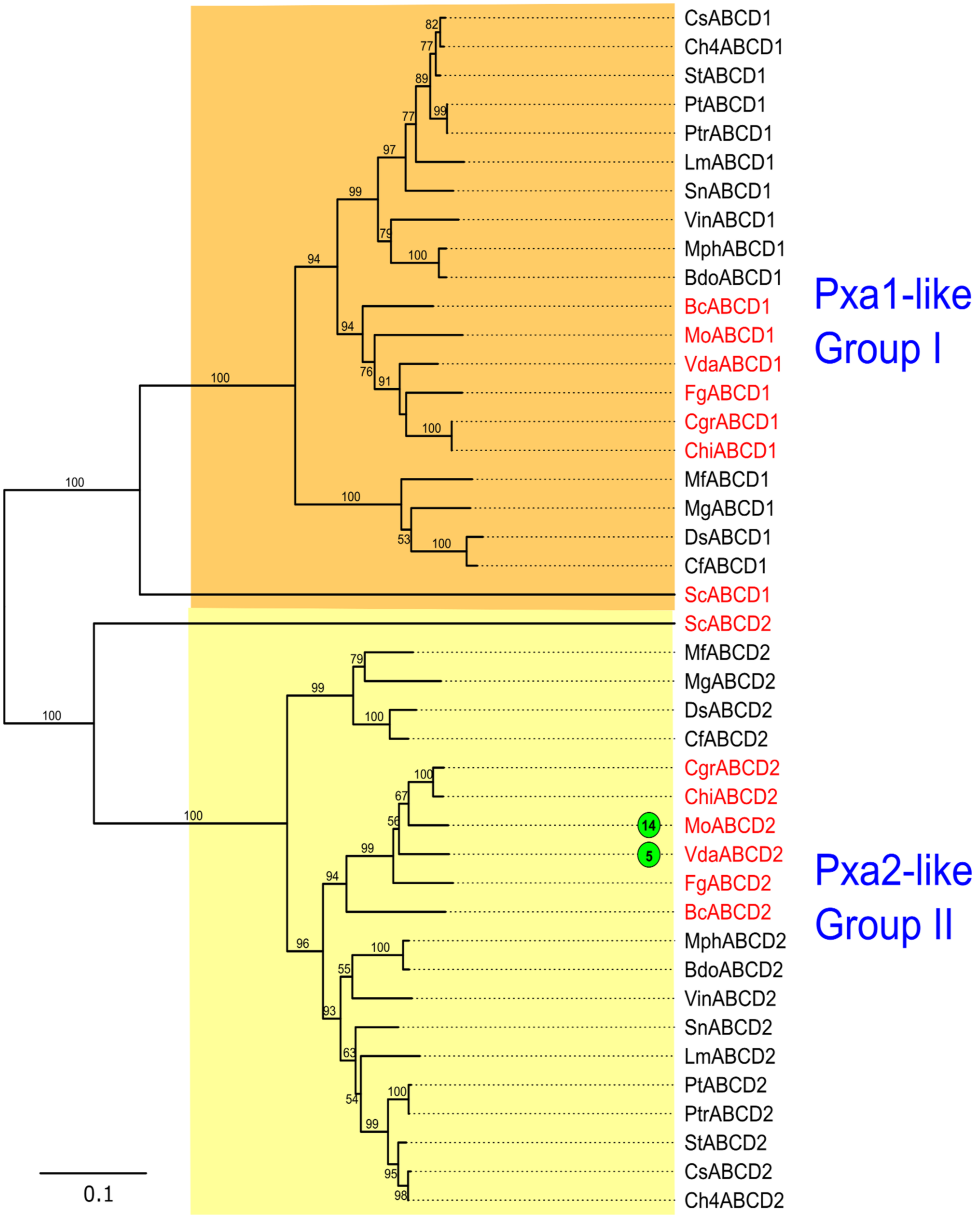
Phylogenetic tree of fungal ABC-D proteins. The tree was generated and annotated as described in case of Figs 4 and 5.

**Fig 9:**
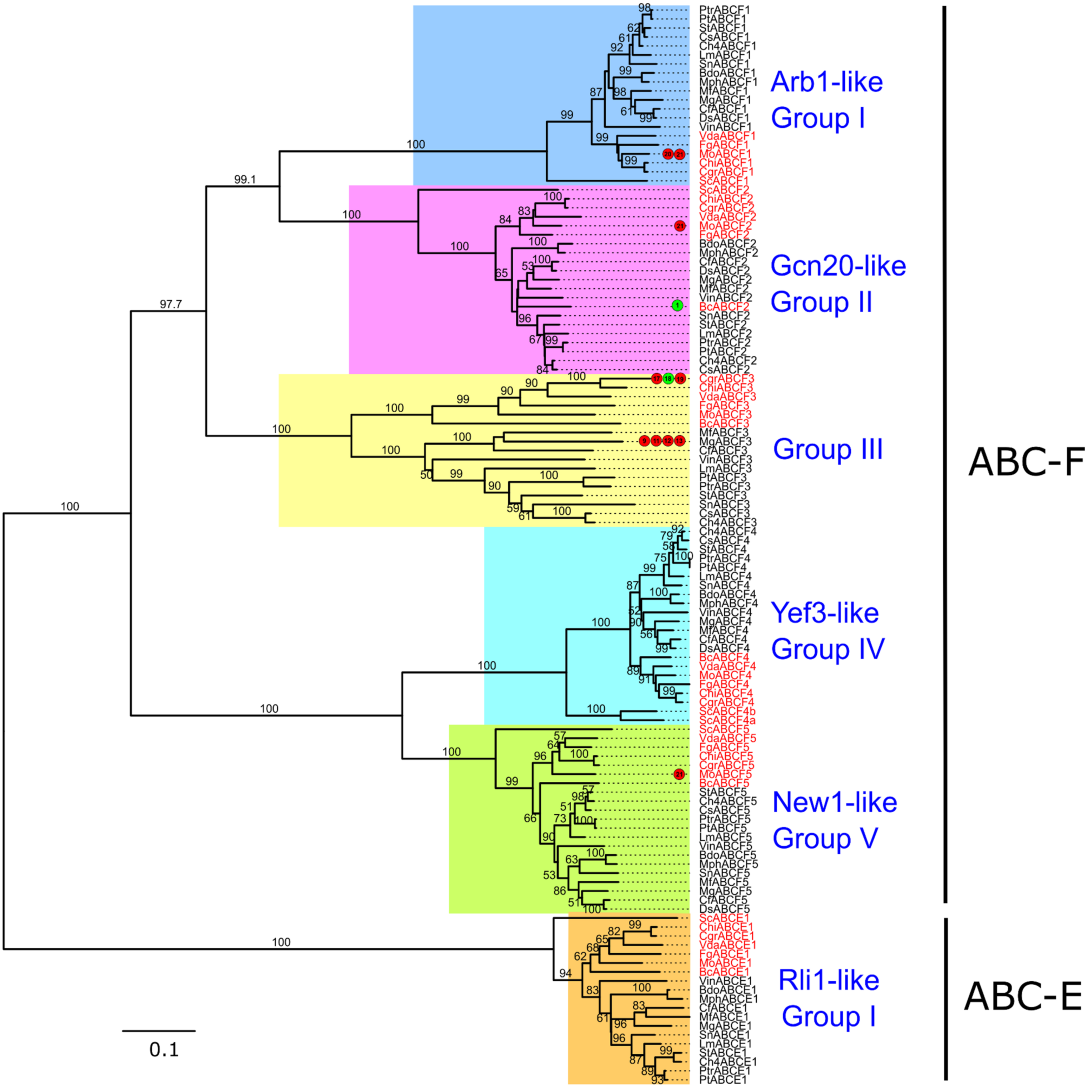
Phylogenetic tree of fungal ABC-E and ABC-F proteins. The tree was generated and annotated as described in case of Figs 4 and 5.

**Fig 10:**
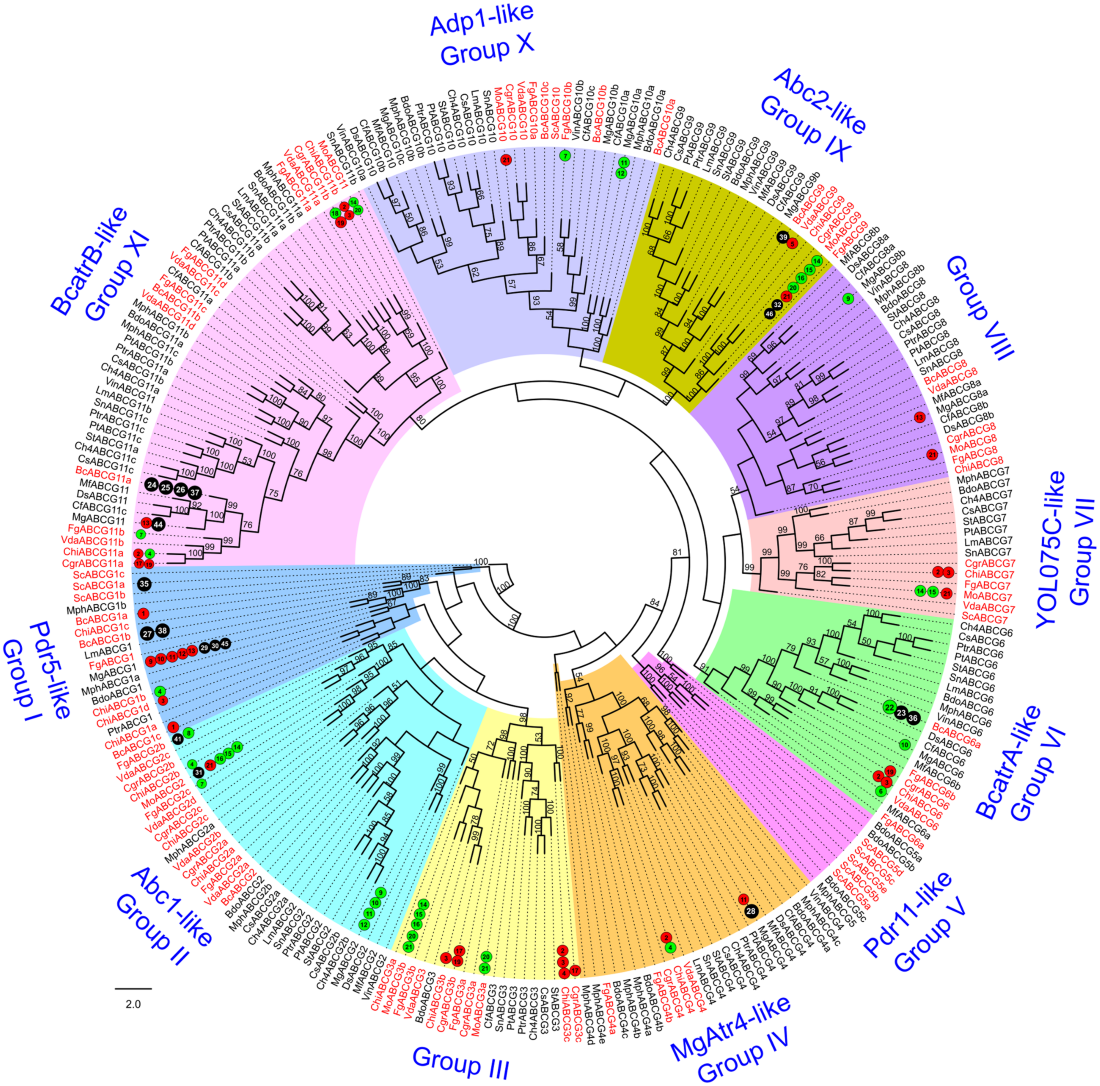
Phylogenetic tree of fungal ABC-G proteins. The tree was generated and annotated as described in case of Figs 4 and 5.

**Fig 11:**
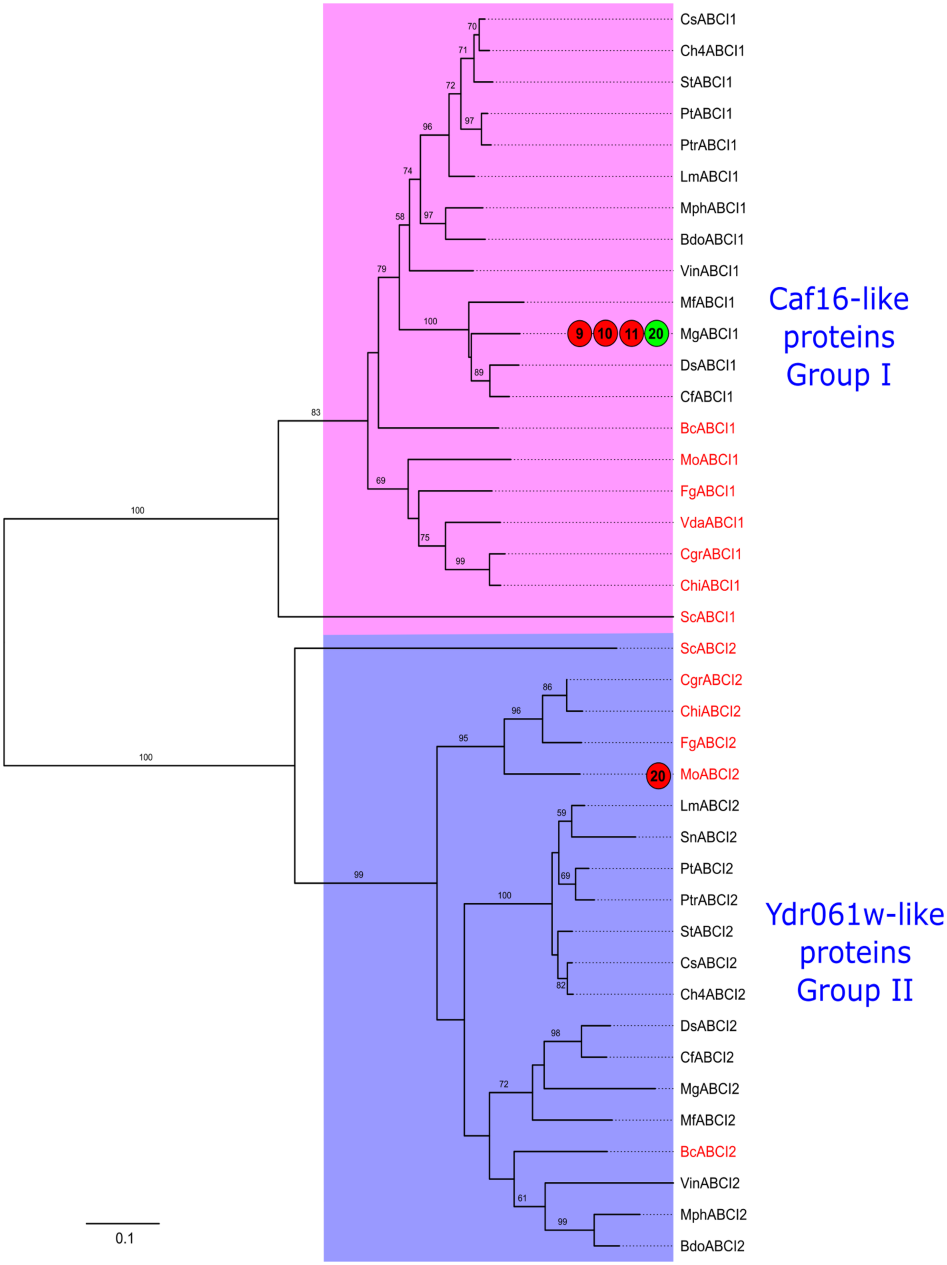
Phylogenetic tree of fungal ABC-I proteins. The tree was generated and annotated as described in case of Figs 4 and 5.

To analyze the phylogenetic relationships among the ABC proteins identified in the present study, multiple sequence alignment (MSA) was performed using ClustalW 2.0.12 [26] and the alignments were manually curated using MEGA v6 [27]. Phylogenetic trees were constructed using all the ABC protein sequences predicted from the Dothideomycetes species (referred to as ‘ABC-Dot’ tree) and separately for individual subfamilies using ABC proteins together from Dothideomycetes and Model species resulting in subfamily trees. Phylogenetic analysis was performed using three methods- Neighbor Joining (NJ), Maximum Likelihood (ML) and Maximum Parsimony (MP). The NJ analysis was performed on our local computational server using PHYLIP v3.69 [28], while the ML analysis was carried out on the CIPRES portal [29] using RAxML v8.1.11 [30]. The MP analysis was performed at MOBYLE 1.5 portal [31] using the PHYLIP-protpars module, except for ABC-Dot and ABC-G trees, which were performed at our local server due to computational limitations at the MOBYLE portal. Using each method, nine trees (ABC-A, BHT, BFT, C, D, E & F, G, I and ABC-Dot) were generated with bootstrap support of 1000 replicates to estimate the reliability of their topologies. The trees were edited and annotated using FigTree v1.4.2 [32], MEGA v6 and Inkscape v0.92 (https://www.inkscape.org). Based on the phylogenetic clustering of ABC proteins from different fungi within each subfamily, the ABC proteins were assigned new IDs in the format- <species abbreviation>ABC<subfamily><group number>‘, which has been used for discussing individual ABC proteins in the subsequent text. The Newick files for the phylogenetic trees (Figs 2–11) are provided in S1–S10 Files.

### Compilation of ABC gene related information from the public domain

Previous literature, databases and gene expression data archives in the public domain were searched for the information related to ABC genes from the fungi involved in the present study. This helped us in compiling a list of ABC genes that were functionally validated for their role in virulence or fungicide tolerance, or differentially expressed in experiments surveying global gene expression during host-pathogen interactions or in response to fungicide treatment. The information on functional analyses were gathered from two sources- Pathogen-Host Interactions Database (PHI-base) [33] and previous literature focused on individual fungal ABC genes. To assess if any of the ABC superfamily members identified in the present study were implicated in virulence or fungicide tolerance, their sequences were compared with those from PHI-base. For this comparison, we used the ABC protein identification and classification method based on InterProScan and BLASTp as described before. As a result, ten ABC proteins from *B*. *cinerea*, *M*. *oryzae*, *M*. *graminicola* and *S*. *cerevisiae* were found in PHI-base that were associated with the following phenotypes- “unaffected pathogenicity”, “reduced virulence” or “loss of pathogenicity” as annotated by the database (S3 Table). Alternatively, peer-reviewed published articles primarily focusing on ABC genes and providing their functions through various experiments were noted (S4 Table). The sequences of such ABC proteins were retrieved, reviewed and classified into subfamilies through the ABC protein identification pipeline as described previously.

A systematic survey of the previous literature on gene expression experiments studying plant-fungal interactions and the response to fungicidal agents, available in the public domain, indicated that they contained valuable expression data on fungal ABC genes. We analyzed the transcriptomics experiments in Ascomycetes fungi, which were carried out using RNAseq, qRT-PCR, microarray or SAGE methods, and extracted the gene expression data for the ABC genes of our interest. To use this information effectively, the gene IDs from the annotations described and the probe IDs from microarray data for all the differentially expressed genes were used for retrieving the nucleotide sequences. These sequences were compared with the ABC protein sequences identified by us for the respective species using BLASTx analysis. In case of RNAseq data, the assembled transcript sequences provided by the respective authors were similarly subjected to BLASTx analysis. The ABC protein identification and classification pipeline using InterProScan as described above was used to further confirm their annotations. The fold change for each of those genes at various time-points in the respective experiments was tabulated (S5 Table).

Additionally, microarray data (GEO Accession- GSE21908) generated by Mathioni *et al*. [34] was re-analyzed to identify *M*. *oryzae* ABC genes differentially expressed *in planta* compared to the mycelia grown in minimal medium (control) (S6 Table). This experiment surveyed the gene expression 72 h post-inoculation of *M*. *oryzae* on *Oryza sativa* (rice) as well as *Hordeum vulgare* (barley). However, the microarray data lacked clear probe annotations. Hence, all the probe sequences were subjected to BLASTx against the *M*. *oryzae* ABC proteins identified earlier in our analysis. The ABC gene-specific probes were further categorized into respective subfamilies. The microarray datasets included four and two replicates respectively for the rice and barley *in planta* experiments and two replicates for the control. The microarray data were examined for expression value distribution and verified as median centered values, confirming that the data were normalized and cross-comparable. Differential gene expression analysis was carried out using the web analytical tool, GEO2R (https://www.ncbi.nlm.nih.gov/geo/geo2r/) [35]. The output p-values were corrected for multiple testing by applying Benjamini and Hochberg false discovery rate method. The ABC genes (represented by respective probes) for which the adjusted p-values were ≤0.05 and fold change ≥2 were considered to be differentially expressed. This analysis revealed 17 and 15 *M*. *oryzae* ABC genes differentially expressed during interactions with rice and barley respectively, and were considered together with the gene expression and functional analyses information curated for *M*. *oryzae* for mapping onto the phylogenetic trees. In all, we compiled a total of 118 ABC genes that showed functionally validated roles during plant-fungal interactions or fungicidal stress, or were differentially expressed during such conditions (S3–S6 Tables). These genes were mapped on the phylogenetic trees, which could hence showcase the evolutionary relationships of the ABC proteins with an overlay of the gene expression and functional roles wherever available.

## Results and discussion

### Identification and classification of fungal ABC proteins

Analysis of protein sequence datasets of the 20 selected fungal species (excluding the reference *S*. *cerevisiae*) revealed 887 ABC proteins, which were then classified into subfamilies A to I (Table 1, S7 and S8 Tables). Apart from these, InterProScan analysis detected 90 ABC protein sequences for which BLASTp indicated considerable similarity with B, C, D or G subfamily members but having incomplete set of domains with only NBD. Since the above mentioned subfamilies characteristically possess transmembrane domain(s) as well, such aberrant sequences could be pseudogenes, anomalies of gene predictions or result of assembly errors or they could also be novel soluble ABC proteins which need to be confirmed by additional experiments. The highest number of such aberrant proteins (19) was found in *C*. *higginsianum*, while in other species the number ranged from one to 15 (S7 Table, column R). These aberrant ABC protein sequences were removed from further analysis. By including the 29 previously characterized ABC proteins from *S*. *cerevisiae*, the list of high confidence ABC proteins totaled to 916, which were included in further analyses (Table 1). Previously, Kovalchuk *et al*. [36] identified 1125 ABC proteins from 28 Basidiomycetes fungi. Their earlier work [10] identified 1109 ABC proteins from 27 fungal species belonging to diverse phyla.

**Table 1:** Number of ABC proteins identified from 21 fungal species and their classification into respective ABC subfamilies.

Sr No.	Category	Species	ABC subfamilies										
			A		B		C	D	E	F	G	I	Total
			HT	FT	HT	FT							
1	**Model**	Saccharomyces cerevisiae*	-	-	3	1	5	2	1	5	10	2	29
2		*Botrytis cinerea*	-	2	3	8	9	2	1	4	12	2	43
3		*Colletotrichum graminicola*	-	2	6	10	13	2	1	5	14	2	55
4		*Colletotrichum higginsianum*	1	2	8	7	16	2	1	5	17	2	61
5		*Fusarium graminearum*	-	1	6	10	15	2	1	5	19	2	61
6		*Magnaporthe oryzae*	-	2	5	12	10	2	1	5	8	2	47
7		*Verticillium dahliae*	-	1	3	5	8	2	1	5	16	1	42
**Total**			**1**	**10**	**34**	**53**	**76**	**14**	**7**	**34**	**96**	**13**	**338**
8	**Test**	*Botryosphaeria dothidea*	-	1	6	10	12	2	1	4	17	2	55
9		*Cladosporium fulvum*	-	1	4	8	7	2	1	5	12	2	42
10		*Cochliobolus heterostrophus*	-	1	3	8	7	2	1	5	12	2	41
11		*Cochliobolus sativus*	-	1	5	10	5	2	-	5	12	2	42
12		*Dothistroma septosporum*	-	1	4	5	6	2	-	4	8	2	32
13		*Leptosphaeria maculans*	-	-	5	8	5	2	1	5	10	2	38
14		*Macrophomina phaseolina*	-	1	6	6	9	2	1	4	19	2	50
15		*Mycosphaerella fijiensis*	-	1	4	7	7	2	1	5	9	2	38
16		*Mycosphaerella graminicola*	1	1	4	3	12	2	1	5	11	2	42
17		*Pyrenophora teres f*. *teres*	-	1	4	8	6	2	1	5	11	2	40
18		*Pyrenophora tritici-repentis*	-	1	4	8	6	2	1	5	11	2	40
19		*Setosphaeria turcica*	-	1	5	7	6	2	1	5	10	2	39
20		*Stagonospora nodorum*	-	2	4	6	9	2	1	5	11	1	41
21		*Venturia inaequalis*	-	1	4	6	10	2	1	5	7	2	38
**Total**			**1**	**14**	**62**	**100**	**107**	**28**	**12**	**67**	**160**	**27**	**578**
**Grand Total**			**2**	**24**	**96**	**153**	**183**	**42**	**19**	**101**	**256**	**40**	**916**

HT- half transporters, FT- full transporters.

*ABC protein reference set retrieved from SwissProt for BLASTp analysis.

In the present study, among the Dothideomycetes fungi, the average number of ABC proteins identified was 41 with the highest number observed in *B*. *dothidea* (55). Considering all the 21 fungi together, the lowest number of ABC proteins was found in *D*. *septosporum* (32) that belonged to Dothideomycetes, while the Sordariomycetes *C*. *higginsianum* and *F*. *graminearum* each possessed the highest number of ABC proteins (61). The detailed information on the identified ABC protein sequences, their BLASTp alignment results, accession IDs and genomic co-ordinates are provided in S8 Table. When the distribution of ABC proteins across the fungal species was considered, we observed that the numbers of subfamily A (1–3), D (2), E (1), F (4–5) and I (1–2) members were smaller and consistent in all these species; whereas the numbers of the other subfamily members were comparatively larger and showed high variation- BHT (3–8), BFT (1–12), C (5–16) and G (7–17) (Table 1). ABC-B, C and G subfamily members were the major contributors to the total number of ABC proteins in each of these individual species. The variability in the number of subfamily B, C and G members in the analyzed fungi could be due to their possible roles in adaptations to varied hosts and lifestyles leading to gene duplications, deletions and variations. On the other hand, subfamily A, D, E, F and I which are known to be involved in fundamental processes of the cell could probably be subjected to comparatively less evolutionary pressure and display conserved gene numbers.

Surprisingly, *C*. *sativus* and *D*. *septosporum* genomes lacked essential ABC-E proteins, while *L*. *maculans* showed the absence of ABC-A proteins, implying that these species might no longer need their functions or could depend on substitute proteins to carry out their roles. Since the present study revealed variation in the numbers of ABC genes across the Dothideomycetes and other Ascomycetes, we examined if it was due to the variation in their genome sizes. The correlation coefficient between the total number of ABC genes from the 21 fungi and their respective genome sizes was found to be insignificant (*r* = 0.2704, p-value = 0.2357, cut-off p≤0.01). The correlation between the total number of predicted genes and the ABC genes was also insignificant (*r* = 0.5438, p-value = 0.0108, cut-off p≤0.01) (S9 Table). Similar investigations [36] on Basidiomycetes ABC protein inventories also revealed that their number and the proteome size or genome size had no significant correlation with each other.

### Phylogenetic relationships among Dothideomycetes ABC proteins and their subfamilies

Phylogenetic analysis involving the 578 ABC protein sequences identified from 14 Dothideomycetes phytopathogens along with 32 reference sequences (29 from *S*. *cerevisiae*; one ABC-A sequence from *A*. *nidulans* and two ABC-A from *D*. *discoideum*) generated the ABC-Dot tree (Fig 3), which represented evolutionary relationships among the Dothideomycetes ABC proteins and their subfamilies. In this phylogenetic tree, clear distinctions were observed among all the subfamily clades. To note, ABC-A and ABC-B subfamily clades were distantly located from each other. ABC-B half transporters (ABC-BHT) and full transporters (ABC-BFT) were clustered in the same major clade with clear distinctions between each other. ABC-C group members were well separated from the ABC-B major clade; however, they were comparatively closer to the ABC-BHT clade. ABC-D clade was located between ABC-E and A clades. ABC-G was divided into three major clades that showed many bifurcations in clade topology. ABC-I (ABCI1, Caf16-like proteins) subclade was located proximal to ABC- F clade. Interestingly, the other ABC-I clade (ABCI2, Ydr061w-like proteins) was clustered with ABC-G clade. Therefore, we suggest that exclusive efforts through additional experiments and sequence characterization should be taken to provide unique identities for both types of ABCI proteins in the ABC protein nomenclature system.

Overall, ABC-B, ABC-C and ABC-G subfamilies displayed considerable diversification in their respective clades while the subfamilies ABC-A, D, E, F and I appeared to be slowly evolving with high sequence conservation. Four distinct groups were observed within the ABC-F clade, where each group showed closely clustered members; implying that the group members should have a conserved function across the analyzed Dothideomycetes genomes. The evolutionary trends observed here were noted through visualization of branch lengths and topologies observed in the ABC-Dot phylogenetic tree. However, exclusive efforts to reveal accurate measures of evolutionary rates among different ABC protein subfamilies are necessary in future studies.

By analyzing the ABC-Dot tree, we could evaluate the assignments of subfamily classification given to the newly identified Dothideomycetes ABC proteins through our annotation pipeline. We anticipated that in the ABC-Dot tree, all the ABC proteins belonging to a particular subfamily would cluster with the reference ABC proteins from the respective subfamily; thus, validating their classification. Accordingly, it was found that all except two ABC proteins were distinctly positioned into the respective subfamilies. This provided us confidence to proceed with subfamily-specific phylogenetic analysis, which could provide better resolution of phylogenetic relationships between the Model and Dothideomycetes ABC proteins at the individual subfamily-level. The two exceptions were: BdoABCBHT1b, being an ABC-B member, was clustered between ABC-E and ABC-D clade, while MphABCD1, an ABC-D protein was located between ABC-BHT and ABC-C clades. Based on the presence of TMD characteristic of the ABC-BHT and D subfamilies and the results of BLASTp analysis, these two proteins were retained in the ABC-C and ABC-G subfamilies, respectively.

### Phylogenetic relationships among Dothideomycetes and Ascomycetes ABC proteins

The ABC protein sequences identified from the Dothideomycetes and Ascomycetes genomes together with those from *S*. *cerevisiae* were divided into subfamilies A, BFT, BHT, C, D, E & F, G and I subsets (Table 1, S8 Table) and individually subjected to phylogenetic analysis (Figs 4–11). For each subfamily, the repertoire of ABC proteins in the fungi analyzed, their phylogenetic relationships together with patterns in gene expression and functional roles of the members in each clade is presented and discussed below.

#### Subfamily A

The ABC-A members participate in biogenesis of lipids, their trafficking and metabolism [37,38]. In humans, variation in their sequence or gene expression have been associated with coronary artery disease, lung surfactant production and ovarian cancer [39–41]. However in fungi, their biological roles have not been well characterized. In our analysis, we found a single ABC-AFT gene in 14 of the 21 species, while five species possessed two ABC-AFTs (Table 1). The maximum number of ABC-AFT (3) was found in *C*. *higginsianum*. *C*. *higginsianum* and *M*. *graminicola* were the only species which also possessed ABC-AHT. Though ABC-A subfamily predominantly includes full transporters, ABC-AFTs have been previously reported [10,42]. Interestingly, we observed that ABC-A proteins were absent in *L*. *maculans* and *S*. *cerevisiae*. The ABC-A phylogenetic tree (Fig 4) showed clear distinction between ABC proteins of Dothideomycetes and those from the other fungi (Model set). As most of the fungal species analyzed in this study exhibited a single ABC-A gene in their genomes, it could be highly possible that their functions are similar. Few examples of characterized ABC-A genes are provided below.

Through an insertional mutagenesis screen, *ABC4* (MoABCA1a/ Locus ID- MGG_00937) of *M*. *oryzae* was identified as essential for appressoria formation and host penetration, whose mutant showed loss of pathogenicity [19]. In addition, increased sensitivity towards the phytoalexin resveratrol and fungicides miconazole and cycloheximide was demonstrated by *ABC4* mutants (Fig 4: node label- MoABCA1a, experiment number 34; S3 Table). This strongly suggested the involvement of this gene in plant-pathogen interactions as well as in fungicide tolerance, making *ABC4* and its orthologs prime candidates for further studies. Furthermore, ChiABCA1a (CH063_04406) in *C*. *higginsianum* was up-regulated during transition from biotrophic to necrotrophic transition phase (60 hpi [hours post-inoculation]), compared to early appressorial pre-penetration phase (22 hpi) and biotrophic phase (40 hpi) of infection with *Arabidopsis thaliana* as host (S5 Table) [43]. This strongly raises the possibility of this ABC-A gene as one of the potential participants in the crucial transition from biotrophic to necrotrophic mode of infection. Further, qRT-PCR analysis revealed that *FgABCA1* (FGSG_08373) was prominently expressed in mycelia of *F*. *graminearum*, harvested at 12 hr post treatment of fungicide Tebuconazole [44], suggesting its sensitivity against azole-based anti-fungals. The expression of *MgABCA1a* (XP_003848920.1) gene of *M*. *graminicola*, a pathogen causing *Septoria tritici* blotch in wheat, was significantly lowered during spore germination (1 dpi [days post-inoculation]), rapid invasive growth (9 dpi) and necrotrophic phase (14 dpi) on wheat leaves, compared to the mycelia from log-phase cultures [45]. Based on the microarray data [46], we also found that *BcABCA1a* (BC1G_11159) of *B*. *cinerea* showed higher expression in dormant spores compared to conidia germinated on apple wax coated surface (S5 Table). Concerted efforts analyzing the role of ABC-A in virulence and fungicide tolerance would be potentially rewarding since our survey noted that the ABC-A members, which were not termed as virulent factors or multidrug/ pleiotropic drug resistance proteins in previous literature, showed gene expression modulation in plant-pathogen interactions and response to fungicides. MfABCA1, DsABCA1, CfABCA1, SnABCA1b and MgABCA1b which are phylogenetically closer to the previously characterized ABC homologs, could be considered for experimental validation of similar roles (S10 Table).

#### Subfamily B

The ABC-B genes are known to encode both full (ABC-BFT) and half transporters (ABC-BHT) that are attributed with high substrate diversity. Several ABC-B proteins have been identified as transporters of pheromones, mitochondrial and antigen peptides, lipid, iron-sulphur (Fe-S) clusters and are also associated with multidrug and heavy metal resistance and detoxification processes ([47–50] and references therein). For example, ABC-B proteins participate in cadmium ion export [51], copper efflux [52], heme formation [53] and securing mitochondria against oxidative stress [47]. In humans, P-glycoprotein (ABCB1/*Pgp*) is the most extensively studied ABC-B, as it is known to efflux various xenobiotics including anti-cancer and anti-microbial drugs [54,55]. To exemplify few roles in plants, the ABC-B proteins are responsible for auxin export, stomatal regulation, Fe homeostasis, Fe-S cluster biogenesis and molybdenum cofactor biosynthesis [56,57]. In fungi, ABC-B transporters identified from *S*. *cerevisiae* are well known for transporting pheromones, mitochondrial peptides or biogenesis of Fe-S clusters [48,49].

In our analysis, the total number of ABC-B genes encoding half or full transporters identified in the Dothideomycetes fungi ranged from 7 (*M*. *graminicola*) to 16 (*B*. *dothidea*) (Table 1, S7 and S8 Tables). Kovalchuk and Driessen [10] and Kovalchuk *et al*. [36] also noted considerable variation in the number of ABC-B genes present among different classes of Ascomycetes and Basidiomycetes species. As the length of full and half transporters were significantly different with average length of ABC-B full transporter being 1306 amino acids and that of half transporter 819 amino acids, their protein sequences were subjected to phylogenetic analysis separately.

Subfamily B full transporters: The 93 ABC-B full transporters identified in the present study, together with three members from *S*. *cerevisiae*, created five groups in the phylogenetic tree (Fig 5). Each group is discussed individually with respect to the members whose genes have previously been characterized functionally, or showed differential expression *in planta* or in response to fungicide treatment, and the grouping of Dothideomycetes ABC proteins with respect to those from the Model fungi.

Group I: In our analysis, Group I was represented by *Ste6* (ScABCBFT1/YKL209C), the only ABC-B full transporter possessed by *S*. *cerevisiae*. It is required for the production and export of A-factor pheromone in yeasts [58]. *Ste6*-like genes were present as one gene per genome in all the fungi analyzed in the present study and in this group, a clear demarcation between Dothideomycetes and non-Dothideomycetes members was seen with *Ste6* positioned as an outgroup. *Ste6* orthologs might be important in survival, virulence or defense mechanisms since Group I appeared to be minimally exposed to gene loss and duplication events. Among the members of Group I, MoABCBFT1 (MGG_04899) showed suppressed gene expression during invasive growth stage (3 dpi) during compatible interactions with both rice and barley compared to growth *in vitro* [34] (S6 Table).

Group II: This group was represented by the multidrug resistance (MDR) gene *Abc3* (MoABCBFT2c /MGG_13762), the loss of which by insertional mutagenesis in *M*. *oryzae* lead to the loss of pathogenicity and impaired the establishment of *M*. *oryzae* during early phase of infection in rice and barley [59]. *Abc3* is thought to counter the host responses of cytotoxicity and oxidative stress and imparts defense against certain xenobiotic conditions encountered during pathogenesis [59]. *Abc3* was also shown to mediate efflux of an endogenous digoxin-like steroidal glycoside which in-turn modulates ion homeostasis and facilitates host invasion during *M*. *oryzae* pathogenesis [60]. Further, in a SAGE experiment, *Abc3* was highly expressed during appressoria formation on plant surface mimicking hydrophobic layer compared to mycelia *in vitro* [61] (S3–S5 Tables), and underlined its important role in early phase of plant pathogenesis. We identified four *Abc3*-like genes in each of the two *Colletotrichum* species and our survey of publicly available data revealed their similar expression patterns *in planta* (Fig 5, Group I). Suppression of their gene expression seemed to be associated with transition from biotrophic phase towards necrotrophic phase of infection (S5 Table). The expression of these genes increased as the disease progressed. We also found that FgABCBFT2b (FGSG_06881) expression was enhanced in the presence of the fungicide Tebuconazole at 12 hr post-treatment compared to untreated controls [44]. In *B*. *cinerea*, *BcABCBFT2a* (BofuT4_P048380.1) was down-regulated in dormant spores, compared to germinating spores [46]. The fungal species considered in our analysis possessed *Abc3*-like genes in the range of two to four, while they were absent in *B*. *dothidea*, *C*. *fulvum*, *D*. *septosporum*, *M*. *graminicola*, *M*. *phaseolina* and *S*. *cerevisiae*. Group I showed very clear distinction between ABC-BFTs from Dothideomycetes and from the Model fungi. Dothideomycetes ABC-BFTs showing stronger orthologous relationships with functionally characterized ABC-BFTs could be associated with their above mentioned roles and considered for further experimental validation (S10 Table). For example, Dothideomycetes LmABCBFT2b could be involved in plant-pathogen interaction and fungicide tolerance similar to MoABCBFT2c (ABC3).

Group III: Group III featured several ABC-BFTs that we could annotate with gene expression data compiled from publicly available sources (Fig 5; S5 and S6 Tables). For example, MoABCBFT3b (MGG_14824) was up-regulated during appressorial development *in vitro* as well as in spore germination-epidermal penetration phase of rice infection by *M*. *oryzae*, compared to germinated spores and *in vitro* mycelia, respectively [61,62]. FgABCBFT3a (FGSG_02786) was significantly expressed in mycelia of *F*. *graminearum* when treated with fungicide Tebuconazole, compared to untreated control [44]. Plant-pathogen interaction studies on *Colletotrichum* species showed that the expression of ChiABCBFT3b (CH063_02770) was markedly suppressed across biotrophic-necrotrophic transition (60 hpi) when compared with appressorial pre-penetration (22 hpi) and biotrophic (40 hpi) phases during interactions with *A*. *thaliana* [43]. In contrast, *C*. *graminicola* infecting maize, significantly up-regulated the expression of CgrABCBFT3a (GLRG_11437) in necrotrophic phase (60 hpi) compared to appressorial pre-penetration (24 hpi) and biotrophic (36 hpi) phases of infection [43]. The above information (S5 Table) suggested that ABC-B members of Group III possibly play a crucial role during plant infection. Group III was divided into two phylogenetic subclades- one comprising mainly Dothideomycetes ABC-BFTs, while the other was a mix of Dothideomycetes and ABC-BFTs from Model fungi, thus providing a good platform to relate the above annotations of characterized ABC-BFTs to Dothideomycetes ABC-BFTs. For example, we could note the ABC-BFTs StABCBFT3a, Ch4ABCBFT3b, CsABCBFT3a, MfABCBFT3a and CsABCBFT3b as candidates for functional characterization related to roles of previously characterized homologs (S10 Table).

Group IV: The *SirA* (LmABCBFT4c /AAS92552.1) gene and their homologs were hosted by Group IV. *SirA* gene disruption has previously shown that it contributes to self-protection against sirodesmin, a non-host-selective epipolythiodioxopiperazine phytotoxin [63]. Previous microarray data revealed that *SirA*-like genes such as MoABCBFT4a (MGG_00141) and MoABCBFT4d (MGG_02348) were co-expressed at significantly higher levels at the invasive growth stage (3 dpi) during compatible interactions between *M*. *oryzae* and rice compared to mycelia *in vitro* [34] (S6 Table). This indicated that such genes might be more active in early stages of pathogenesis. In another experiment, expression of FgABCBFT4b (FGSG_11988) was significantly down-regulated in the presence of the fungicide Tebuconazole [44]. Among the fungal genomes we analyzed, *SirA* homologs were found in range one to five (*M*. *oryzae*). The *SirA* homologs were absent in the genomes of *M*. *graminicola*, *S*. *cerevisiae* and *V*. *dahliae*. When we analyzed the topology of this phylogenetic tree, no clear-cut distinctions between Dothideomycetes and others were seen. Here, *SirA* being one of the few well-studied Dothideomycetes ABC-BFTs, aided in annotating members of its own class as well as non-Dothideomycetes ABC-BFT genes and presented us five ABC genes with potential for further functional characterization (S10 Table).

Group V: From Group V, we could trace few ABC-BFTs with up-regulated gene expression from the public data related to pathogenesis and fungicide tolerance. In an *in planta* study involving *Colletotrichum* species, ChiABCBFT5 (CH063_11654) was highly expressed in necrotrophic conditions (60 hpi) compared to appressorial pre-penetration (22 hpi) as well as biotrophic phase (40 hpi), while its ortholog CgrABCBFT5 (GLRG_08994) was prominently up-regulated in biotrophic phase compared to appressorial developmental phase [43]. In the present study, *B*. *dothidea* revealed five genes of this group, while some of the fungal genomes possessed genes ranging from none to three. The phylogenetic tree of this group revealed two subclades—one of the clades contained Model ABC-BFTs along with Dothideomycetes ABC-BFTs that could likely be involved in roles similar to those of the above mentioned previously characterized ABC-BFTs (S10 Table).

Subfamily B half transporters: The fungal ABC-B half transporters are present either in mitochondrial or vacuolar membranes. The ABC-BHTs characterized so far are exporters of mitochondrial peptides, Fe-S clusters and heavy metal ions [64,65]. The phylogenetic tree of ABC-BHT in this study featured five distinct groups (Fig 6).

Group I: This was a smaller outgroup of Group II and contained only five ABC-BHTs, out of which ChiABCBHT1a was suppressed during transition from biotrophic to necrotrophic phase (60 hpi) compared to appressorial pre-penetration phase (22 hpi) (S5 Table) [43], suggesting that this gene was more active in early stages of fungal infection.

Group II: It was represented by two mitochondrial ABC-B transporters, *Mdl1* (ScABCBHT2a/ YLR188W) and *Mdl2* (ScABCBHT2b/ YPL270W) of *S*. *cerevisiae*. Of these, *Mdl1* exports mitochondrial peptides through its inner membrane and also plays role in conferring cellular resistance towards oxidative stress (S2 Table) [66], while the role of *Mdl2* still remains obscure. Apart from *S*. *cerevisiae* possessing two such genes, one *Mdl1*-like gene was present in all the species analyzed in the present study. The phylogenetic tree showed ScABCBHT2a/2b as outgroups, while the rest of the group showed clear distinction between the Dothideomycetes ABC proteins and those from the other fungi (except BcABCBHT2). More studies are needed to annotate a generalized function for this group.

Group III: *Atm1* (ScABCBHT2) of *S*. *cerevisiae*, the representative of Group III BHTs is a mitochondrial protein involved in biogenesis and transport of Fe-S clusters required for iron homeostasis and is necessary for normal cellular growth [48,67,68]. Orthologs of this gene were present in all the fungal genomes that we analyzed, except in *S*. *nodorum*. Schaedler *et al*. [47] suggested that *Atm1* and *Mdl1* may have overlapping substrate specificities and hence this possibility might help species like *S*. *nodorum* lacking *Atm1*-like genes to compensate their loss with the help of *Mdl1*-like genes. Group III showed a cluster of non-Dothideomycetes ABC-BHT members flanked by Dothideomycetes ABC-BHTs; however *Atm1* (ScABCBHT3) clustered distinctly from the rest of the members of this group. This group did not possess functional information on any of the genes from the phytopathogens we studied and hence need to be surveyed for *Atm1* and *Mdl1*-like functions.

Group IV: Group IV members were present in all the fungal genomes analyzed in the present study except in *S*. *cerevisiae*. Interestingly, one of the group members, MoABCBHT4 (MGG_05190) was significantly up-regulated during appressoria formation *in vitro* [61] as well as during invasive growth stage (3 dpi) in compatible interactions with rice compared to mycelia *in vitro* [34] (S5 and S6 Tables). Conversely, VdaABCBHT4 (VDAG_04079) of *V*. *dahliae* was over-expressed in microsclerotia forming stage compared to conidia forming stage *in vitro* [69]. Group IV showed clear distinction between Dothideomycetes and the other ABC-BHTs. As an exception, CsABCBHT4b and BdoABCBHT4 were closely grouped with the ABC-BHTs from Model species. Hence, we propose that the above annotations could be more associable to these two members as compared to the rest of the members in this group (S10 Table).

Group V: In Group V, the fungal genomes analyzed by us represented one to three ABC proteins however, *B*. *cinerea*, *S*. *cerevisiae*, *C*. *heterostrophus* and *V*. *dahliae* showed none. This group lacked well-characterized members and hence mapping of gene expression information onto the phylogenetic tree provided valuable insights into their possible roles in fungal defense strategies. We observed that CgrABCBHT5b (GLRG_11556) was up-regulated during advanced stages of *C*. *graminicola* infection in maize [44]. In addition, ChiABCBHT5b (CH063_04365) was down-regulated during biotrophic-necrotrophic transition phase occurring at 60 hpi on *A*. *thaliana* leaves, compared to biotrophic (22 hpi) and appressorial (40 hpi) stages [43]. However, the phylogenetic tree showed clear separations between the ABC-BHTs from the Model and Test fungi.

#### Subfamily C

Members of ABC-C subfamily are full transporters [8,10,50]. Most of the ABC-C proteins in eukaryotes are localized in the vacuolar membrane and engage in sequestration and detoxification of xenobiotic toxic substances, function as chloride ion channel, provide heavy metal resistance and export conjugates of organic anion molecules like glutathione/ glucuronide ([70–73] and references therein). In humans, mutation in the *SUR1* (sulfonylurea receptor 1, ABCC8) gene causes neonatal diabetes [74]. Similarly, ABCC1, ABCC3, ABCC4 are attributed with cytotoxic drug efflux and can also interfere during chemotherapeutic treatments of cancer like neuroblastoma [75]. In plants, ABC-C proteins aid in maturation of fruits [76], arsenic tolerance [77], chlorophyll degradation [78] and regulation of ion channel of guard cell plasma membranes [79]. Most of the fungal ABC-C genes are related to heavy metal and metalloid resistance, bilirubin, bile acid, oligomycin or nucleoside transport and fungicide resistance [10]. Kim *et al*. [80] provided valuable information on the expression of 11 ABC-C genes in *M*. *oryzae* under various biotic and abiotic stresses, wherein they indicated that differentially up-regulated ABC-C genes were mostly found in association with abiotic stress conditions compared to biotic ones. In the present study, the numbers of ABC-C transporter genes ranged from 5 to 16 in the analyzed genomes, suggesting that these fungi might show variation in the number of ABC-C according to their lifestyle or host-specificity (Table 1). Phylogenetic analysis of the ABC-C identified seven distinct groups (Fig 7).

Group I: *MoABC5* (MoABCC1/MGG_05009) was the representative of Group I. *MoABC5* knockouts showed reduced virulence on rice [80] and *MoABC5* was over-expressed eight folds during invasive growth stage on rice *in planta* compared to mycelia *in vitro* (Tale S6) [34]. Our efforts to compile the results of various gene expression experiments from the public domain provided valuable information on Group I members that were not evident before (S5 Table). *F*. *graminearum* FgABCC1d (FGSG_00046) was significantly up-regulated in response to the fungicide Tebuconazole, making it a good candidate for studying anti-fungicidal responses [44]. ABC-C genes of *C*. *higginsianum* showed reduced gene expression post infection on *Arabidopsis*. ChiABCC1d (CH063_06043) and ChiABCC1c (CH063_03724) were significantly down-regulated during biotrophic and biotrophic-necrotrophic transition phases respectively, compared to the appressorial pre-penetration phase. Whereas, gene expression of ChiABCC1e (CH063_03641) was also suppressed in both the above conditions and during biotrophic-necrotrophic transition stage compared to biotrophic phase *in planta* [43]. Similarly, in *C*. *graminicola* CgrABCC1c (GLRG_05467) showed significantly lowered gene expression during biotrophic phase, compared to appressorial pre-penetration phase in maize [43]. Further, FgABCC1b (FGSG_02316) was highly expressed in *F*. *graminearum* hyphae growing inside wheat coleoptiles [81] and thus suggested that this ABC-C gene might aid this fungus while establishing in the host. The Group I clade showed many small, discretely located subclades consisting of a mix of Model and Dothideomycetes ABC-C members. Every such subclade had few of the annotated ABC-C members, whose functions could be associated to closely clustered Dothideomycetes ABC-C proteins. We could note six of such Dothideomycetes ABC-C members which can be included for experimental validation (S10 Table).

Group II: This group contained three functionally characterized ABC-C genes *viz*., FgABC1 (FgABCC2a/ FGSG_10995), FgABC4 (FgABCC2c/ FGSG_17058) and MoABC6 (MoABCC2/ MGG_05044) (S4 Table). Deletion mutants of *FgABC1* showed reduced virulence towards wheat, barley and maize; while *FgABC4* deletion mutants were sensitive towards triazoles and fenarimol. *FgABC1* might be responsible for secretion of fungal secondary metabolites, which in-turn could facilitate host defense [18]. Additionally, *FgABC1* and its paralog *FgABC4* showed significant increase in transcript levels when treated with the fungicide Tebuconazole, where *FgABC4* showed higher expression compared to *FgABC1* (S5 Table) [44]. In *M*. *oryzae*, *MoABC6* played a major role during various abiotic stresses such as salt stress and nutrient deficient conditions [80]. *MoABC6* was also highly suppressed during appressorial development on plant surface mimicking hydrophobic condition compared to mycelia *in vitro* (S6 Table) [61]. Additionally, in this group, ABC-C genes showed expression modulation during the infection stages of *Colletotrichum* spp. The transcript levels of *C*. *higginsianum* ChiABCC2a (CH063_02962), ChiABCC2c (CH063_02722) and ChiABCC2d (CH063_01027) were significantly suppressed during biotrophic-necrotrophic transition stage (60 hpi) compared to biotrophic and appressorial pre-penetration stage (22 hpi) in *Arabidopsis* leaves [43]. In the same study, ChiABCC2a gene expression was down-regulated along with ChiABCC2b (CH063_01081) during biotrophic-necrotrophic transition stage compared to biotrophic phase expression levels. Furthermore, CgrABCC2a (GLRG_06008) of *C*. *graminicola* had higher expression across necrotrophic phase (60 hpi), compared to biotrophic phase (36 hpi) during interactions with maize [43].

During appressorial development of *M*. *oryzae*, the expression of MoABCC2 (MGG_05044) was down-regulated compared to mycelia *in vitro* (S5 Table) [61]. The above examples suggested that ABC-C genes of this group played roles in pathogenesis and fungicide tolerance by modulating their expression as required. Among the fungal genomes that we analyzed, the numbers of Group II ABC-C genes showed large variation ranging from one to five. In contrast, such genes were absent in *C*. *sativus*, *D*. *septosporum*, *L*. *maculans*, *P*. *teres*, *P*. *tritici-repentis*, *S*. *cerevisiae*, *S*. *turcica* and *V*. *inaequalis*. This suggested that these genes might have undergone several gene duplication and deletions events, which might be due to their roles in adaption to the host environment or developing defense and attack strategies against their respective hosts. Group II did not show clear separations between Dothideomycetes and non-Dothideomycetes members and few small clusters comprising both the types were seen, though such clusters were dominated by annotated ABC-C members. Being surrounded by Model ABC-C members, it would be easier to extend their functions to the newly identified Dothideomycetes ABC-C members and characterize them in detail. Some of the members of Group II that we noted for experimental validation include- MgABCC2b and MfABCC2b (S10 Table).

Group III: Yor1 (ScABCC3/YGR281W) was the representative ABC-C of Group III. This ABC-C protein is the only ABC-C transporter of yeast that is localized to the plasma membrane, the rest four being vacuolar transporters. Yor1 is a multidrug transporter and can confer resistance to antibiotics like oligomycin and doxorubicin [71,82], and also can transport heavy metals like cadmium [70]. The genes from this group were identified in all the fungal genomes that we analyzed, with up to three genes in *M*. *graminicola*. Six genes corresponding to the members of this group showed expression modulation in conditions associated with host-pathogen interactions (S5 and S6 Tables). Of these, notably *M*. *graminicola* MgABCC3a expression was suppressed *in planta* throughout different phases of plant infection i.e. spore germination stage (1 dpi), slow symptomless invasive growth (4 dpi), rapid invasive growth (9 dpi), necrotrophic phase (14 dpi) and extreme necrotrophic phase (21 dpi), compared to that in mycelia from log-phase cultures [45]. In *M*. *oryzae*, MoABCC3 (MGG_08309) was down-regulated across invasive growth stage (3 dpi) during compatible interactions with barley [34]. Significant up-regulation of *F*. *graminearum* FgABCC3b in mycelia treated with the fungicide Tebuconazole was confirmed by qRT-PCR analysis [44]. Interestingly, Group III did not show clear distinction between the Dothideomycetes and ABC-C proteins from the other classes. Yor1, in spite of being outgroup, remained closely clustered with ABC-C such as DsABCC3a and MgABCC3a/3c which could be evaluated for Yor1-like functional roles.

Group IV: Group IV had two reference ABC-C from *S*. *cerevisiae*, namely *Ycf1* (ScABCC4a/ YDR135C) and *Bpt1* (ScABCC4b/ YLL015W). *Ycf1* encodes a glutathione S-conjugate transporter [83] and mediates cadmium resistance through vacuolar sequestration in yeasts [72]. In addition to the transport of glutathione S-conjugates, Bpt1 also transports bile and Ade2 pigments and plays a role in cadmium detoxification [84]. It has been shown that Ycf1 and Bpt1 co-operate for vacuolar transport of free, unconjugated bilirubin in *S*. *cerevisiae* [85] and both act as positive regulators of vacuole fusion [73]. Orthologs of these genes were present in all the fungal species that we analyzed, except in *B*. *dothidea*. Among the Group IV members, MoABC5 (MoABCC4b/ MGG_05009) was up-regulated during appressorial development phase compared to mycelia *in vitro* in a SAGE experiment (S5 Table) [61]. Also, the expression of *V*. *dahliae* VdaABCC4b was high in microsclerotia developing cultures (10 dpi) compared to non-microsclerotia forming cultures [86]. These examples strongly suggest the importance of such ABC-C proteins in fundamental cellular processes and also during plant-pathogen interaction as evident from the gene expression data. The clade of Group IV consisted of Dothideomycetes ABC-C flanked by subclades of ABC-C from the Model species.

Group V: Group V contained one ABC-C gene each from 18 of the analyzed fungal genomes, two from *V*. *inaequalis* and none from *S*. *cerevisiae* and *B*. *cinerea*. No clear demarcation between the members of Dothideomycetes and the others was observed in the phylogenetic tree. However, only one member namely, MoABCC5 (MGG_07567) showed significant down-regulation during *in planta* interactions with barley in invasive growth conditions compared to its growth *in vitro* (S6 Table). [34] Hence, targeted studies are needed to characterize this group in detail.

Group VI: In the fungal genomes analyzed in the present study, one to three ABC-C proteins were present in Group VI. Our literature data mining revealed that MoABCC6a (MGG_03736) of *M*. *oryzae* was up-regulated in invasive growth stage during rice infection; while surprisingly its paralog MoABCC6b (MGG_05746) was down-regulated in the same experimental conditions (S6 Table) [34]. Additionally, the expression of MoABCC6a was higher during appressorial development phase of *M*. *oryzae* after its exposure to *in vitro* plant mimicking hydrophobic surface (S5 Table) [61]. Hence, MoABCC6a could be an important factor during early stages of pathogenesis. The phylogenetic clade of this group showed separation between Dothideomycetes and Model fungi, except that BcABCC6b and MoABCC6b were clustered with the Dothideomycetes members. As the Dothideomycetes ABC-C transporters, VinABCC6, BdoABCC6a and MphABCC6b clustered with the characterized Group VI members, we propose them as promising targets for experimental validation for roles in early host invasion (S10 Table).

Group VII: Group VII contained *S*. *cerevisiae*, Vmr1 (ScABCC7a/YHL035C) and Ybt1 (ScABCC7b/ YLL048C). *Vmr1* encodes evolutionarily well-conserved yeast MRP located in the vacuolar membrane. It is a glutathione S-conjugate transporter and confers tolerance against chemicals such as cycloheximide, 2,4-dichlorophenoxyacetic acid, 2,4-dinitrophenol and metal resistance towards cadmium and mercury [87]. Ybt1 is a bile acid transporter and regulates the vacuolar import of phosphatidylcholine, carries out efflux of lumenal calcium stores and acts as a negative regulator of vacuolar fusion process [88,89]. In our survey of public data, we found that *M*. *oryzae* MoABC7 (MoABCC7/ MGG_04855) when knocked down, produced significantly reduced conidiation, indicating its role in early stages of pathogenesis (S4 Table). In addition, MoABC7 was up-regulated when treated with iprobenfos and isoprothiolane, which inhibit fungal choline biosynthesis; suggesting its additional role in efflux of toxicants [80]. In another experiment, this ABC-C transporter was up-regulated in invasive growth phase (3 dpi) of rice infection cycle (S6 Table) [34]. Group VII showed separation between subclades of ABC-C from Model and Test species, where MphABCC7a and ScABCC7a/b positioned as outgroup to both the subclades; which might indicate their conserved and less evolving nature than the rest of the group members.

#### Subfamily D

The members of ABC-D subfamily are half transporters localized in peroxisomes [90]. Their functions are evolutionarily conserved and they are involved in long-chain fatty acid (FA) transport and metabolism [91]. The two ABC-D proteins in *S*. *cerevisiae*, Pxa1 and Pxa2, are heterodimers that interact with each other to carry out the import of long-chain FA into peroxisomal matrix and thereby participate in their beta-oxidation [91]. One *Pxa1*-like and *Pxa2*-like gene each was identified in all the fungal genomes analyzed in the present study (Table 1). Phylogenetic analysis of ABC-D proteins revealed two different groups (Fig 8), similar to those found by Kovalchuk and Driessen [10], each represented by Pxa1 and Pxa2 respectively. When we compiled the gene expression and functional analysis data for this subfamily, we found that in *M*. *oryzae*, it has been shown that the absence of peroxisome biogenesis in appressoria causes complete loss of pathogenicity, and therefore peroxisomal beta-oxidation is essential for both appressorium function and subsequent pathogen proliferation on rice leaves [92]. We found that *M*. *oryzae* MoABCD2 (MGG_06707; homolog of *S*. *cerevisiae* Pxa2) was up-regulated during spore germination-appressorial differentiation stage *in vitro* conditions, compared to that in mycelia [61] (S5 Table). This indicated that there is a possibility that MoABCD2 is an important gene linking appressorial development, peroxisomal metabolism and infection strategies of this fungus. In another experiment, VdaABCD2 was up-regulated during microsclerotia formation compared to non-microsclerotia forming culture [86], suggesting its importance fungal survival. In both the groups of this subfamily, most of the Dothideomycetes members were clustered separately from the rest, while ABC-D members from *M*. *fijiensis*, *D*. *septosporum*, *C*. *fulvum* and *M*. *graminicola* formed outgroups.

#### Subfamilies E and F

The proteins of both these subfamilies consisted of only NBD and lacked TMD and all except MgABCF1 contained two NBDs each. Hence, these two subfamilies were considered together for phylogenetic analysis; which aided in better understanding of their relationships. Previously, Kovalchuk and Driessen [10] also subjected these two subfamilies together for phylogenetic analysis.

As ABC-E members are soluble proteins lacking TMDs, they are possibly not involved in transmembrane transport of molecules [93]. ABC-E proteins have been identified in the genomes of prokaryotes as well as eukaryotes and exhibit high sequence similarity within the fungal kingdom and across the eukaryotes [10,36]. Our study also confirmed this high conservation, both in terms of the number of ABC-E genes possessed (Table 1) by each considered species and sequence similarity (>90%) among them. This supports the notion that the biological function of ABC-E proteins could also be highly conserved. The most well-known member of this subfamily is the *S*. *cerevisiae* Rli1 (ScABCE1), that promotes translation pre-initiation complex assembly [94]. It is also involved in ribosomal subunit maturation, ribosomal subunit transport to the cytoplasm, ribosome recycling, translation (re)initiation through interaction with the eukaryotic initiation factor 3 (eIF3) complex as well as in translation termination [95,96]. Alhebshi *et al*. [97], showed that Rli1 plays a role in oxidative stress resistance, and therefore it would be worth to investigate whether ABC-E members could protect the fungal cells during oxidative stress in plant-pathogen interactions. Alternatively, fungal ABC-E proteins might be the potential targets of antifungal compounds released by the hosts that could suppress their anti-oxidative property and expose the fungal cells to oxidative stress.

In the present study, all the analyzed fungal genomes possessed one ABC-E gene each, except *D*. *septosporum* and *C*. *sativus* that possessed none (Table 1). Dothideomycetes and non-Dothideomycetes ABC-E members were clustered in distinct subgroups, though both formed a closely clustered clade (Fig 9). Moreover, none of the genes encoding ABC-E proteins were found showing expression modulation or functional roles during fungal interactions with hosts or upon exposure to fungicides.

The ABC-F members are implicated in fundamental cellular processes like ribosome biogenesis, mRNA export, translation initiation and elongation and also act as elongation factors ([93,98] and references therein). Several new roles of ABC-F genes have been discovered recently. For instance, the bacterial Vga(A) is one of the antibiotic resistance associated (ARE) ABC-F proteins that determines antibiotic resistance specificity [99], while its homolog EttA controls translation through a mechanism sensitive to cellular energy status [100]. Recently, the translational regulators ABCF3 and GCN-1 proteins of *C*. *elegans* were also shown to be interacting together for promoting apoptosis [101]. However, the roles and functions of ABC-F in fungi especially in Dothideomycetes largely remain unknown. Hence, our study provides a very good starting point to elucidate the function of ABC-F proteins of this class. ABC-F proteins were present in all the 21 analyzed fungal genomes. Seventeen of these fungi possessed sets of five ABC-F genes each, while four others *viz*. *D*. *septosporum*, *M*. *phaseolina*, *B*. *cinerea* and *B*. *dothidea* possessed a set of four ABC-F members each. In all, 67 ABC-F proteins were identified from Dothideomycetes species and 34 from Model species (Table 1). The phylogenetic analysis categorized them into five distinct groups (Fig 9).

Group I: Group I contained the reference Arb1 (ScABCF1) of *S*. *cerevisiae*, which is essential for cell survival and promotes 40S and 60S ribosome biogenesis in yeast [98]. Except *B*. *cinerea*, all the species that we analyzed possessed a single *Arb1*-like gene. Our analysis of the publicly available microarray data revealed that the gene expression of MoABCF1 (MGG_11862) of *M*. *oryzae* was suppressed during compatible interaction with rice as well as with barley at 72 hpi during invasive growth phase compared to mycelia *in vitro* (S6 Table) [34]. As down-regulation of MoABCF1 was seen in both rice and barley, it might be a common target of plant defense molecules that might lead to disturbance of fundamental process like ribosome biogenesis in the fungi, thus creating opportunities for the plant to overcome the infection. The phylogenetic clade of this group was clearly divided into ABC-F members from Dothideomycetes and from the Model fungi.

Group II: Group II contained *S*. *cerevisiae* Gcn20 (ScABCF2), which acts as a positive regulator of Gcn2p kinase activity, both of which in turn control GCN4 that regulates the genes involved in amino acid biosynthesis [102,103]. A single *Gcn20*-like gene was found in each of the fungi considered in the present study. Our compilation of the publicly available microarray data showed that in *B*. *cinerea*, the expression of *Gcn20*-like BcABCF2 (BofuT4_P101380.1) gene was higher in dormant spores compared to germinating conidia on apple wax coated surface (S5 Table) [46]. Our re-analysis of the microarray data GSE21908 showed that the expression of MoABCF2 (MGG_11547) was significantly lowered in *M*. *oryzae* during 72 hpi on barley compared to mycelia *in vitro* (S6 Table) [34]. The phylogenetic relationships in Group II showed the separation between subclades of Dothideomycetes and ABC-F from the Model fungi. As an exception, BcABCF2, a non-Dothideomycetes ABC-F protein was closely clustered with the Dothideomycetes ABCF proteins. Thus, VinABCF2, MfABCF2, MgABCF2 and SnABCF2 could be evaluated for similar roles related to BcABCF2 (S10 Table).

Group III: We found that 17 of the 21 species analyzed by us possessed a single ABC-F gene of Group III. However, *M*. *phaseolina*, *D*. *septosporum*, *S*. *cerevisiae* and *B*. *dothidea* did not harbor any gene of this group. Though this group did not include any member that was previously characterized functionally, our examination of previous literature revealed experiments involving Dothideomycetes phytopathogens that contained gene expression data on Group III ABC-F members. During *M*. *graminicola* infection on wheat, MgABCF3 was down-regulated during spore germination (1 dpi), rapid invasive growth (9 dpi), necrotrophic phase and sporulation onset (14 dpi) and extreme necrotrophic phase (21 dpi) compared to mycelia from log-phase cultures [45]. Additionally, in *C*. *graminicola* inoculated on maize leaves, the transcript levels of CgrABCF3 (GLRG_06093) were higher in appressorial pre-penetration stage and reduced in biotrophic phase and again peaked in the necrotrophic stage (S5 Table) [43]. We found this interesting as this putative elongation factor showed modulation in its gene expression across different stages of host invasion in the pathogenic cycle. The phylogenetic topology of this group was seen with clear division into Dothideomycetes and ABC-F from Model fungi. Notably, we could annotate a member each from both Dothideomycetes and Model species with expression data in the context of host-pathogen interactions which would be useful for characterizing ABC-F from both the fungal sets.

Group IV: This group was represented by two reference proteins from *S*. *cerevisiae*, i.e. Yef3 (ScABCF4a) and Hef3 (ScABCF4b). Yef3 is a translation elongation factor, which is essential for viability of *S*. *cerevisiae* cells along with Arb1 (ABC-F, Group I) and Rli1 (ABC-E) [104]. Hef3 is related to Yef3 but it encodes a non-functional homolog of Yef3 [105]. A single *Yef3*-like gene was present in all the fungal genomes analyzed by us, highlighting its importance through conservation in terms of the number and sequence composition in the analyzed fungi. Clear division was seen between the ABC-F proteins from the Model and Dothideomycetes fungi. Further in this group, *S*. *cerevisiae* ABC-F proteins positioned themselves as clear outgroup to ABC proteins from Dothideomycetes and Model fungi.

Group V: In Group V, each of the fungi in the present analysis was represented with a single ABC-F member. The representative member of this clade was *S*. *cerevisiae* New1 (ScABCF5), which has been reported to possess a regulatory role in prion state formations [106]. Our analysis revealed that *M*. *oryzae* MoABCF5 (MGG_02572) was significantly down-regulated during invasive growth phase at 72 hpi on barley compared to mycelia *in vitro* (S6 Table) [34]. Though this group also showed clean demarcation between the Model and Dothideomycetes ABC-F, *B*. *cinerea* ABC-F member BcABCF5 was closely clustered with the Dothideomycetes ABC-F members.

#### Subfamily G

ABC-G subfamily proteins are very well-studied ABC transporters. One of the most notable examples is the wheat ABC-G gene *Lr34*, which confers resistance to multiple fungal pathogens. Heterologous expression of this gene in barley provided multi-pathogen resistance to this crop [107,108]. In *Arabidopsis*, ABCG32 and ABCG13 are required for leaf development and flower cuticle secretions respectively [109,110], while Atwbc19 confers resistance against kanamycin [111]. Further, it has also been observed that the plant ABC-G transporters play roles in leaf water retention [112], shoot branching [113] and transport of various phytohormones [114]. In animals, the genes encoding ABC-G half transporters carry out transport of drugs, toxins, lipids or secondary metabolites ([115] and references therein). It was also indicated that ABC-G transporters might have an important role in mouse brain development, presumably by regulating and maintaining lipid homeostasis [116]. The human genes like ABCG1, ABCG5 and ABCG8 are known for regulation of lipid trafficking [117]. In fungi, the PDR ABC-G proteins protect them against harmful cellular metabolites and toxins, carry out sterol transport, translocate membrane phospholipids, aid in ion exchange and are associated with quorum sensing in yeast ([118] and references therein). The fungal ABC-G proteins also play roles in plant pathogenesis by generating resistance towards host phytoalexins and/or by acting as virulence factors [119–121].

We identified 256 ABC-G proteins in 21 fungal species ranging from seven in *V*. *inaequalis* to 19 in *M*. *phaseolina* (Table 1). We found as many as 19 ABC-G gene knockout/ mutant based studies related to pathogenicity or fungicide tolerance for the fungi considered in the present study (S3 and S4 Tables). Phylogenetic analysis of ABC-G by Kovalchuk and Driessen [10] showed seven groups, whereas, the phylogenetic tree for the ABC-G identified in the present study (Fig 10) showed 11 distinct groups which are discussed individually below.

Group I: This group contained functionally characterized ABC-G genes from *B*. *cinerea*, *M*. *graminicola*, *S*. *cerevisiae*, *F*. *graminearum* and *C*. *higginsianum* (S3 and S4 Tables). The highest number of Group I members were present in *C*. *higginsianum* (4). This group interestingly had three ABC proteins from *S*. *cerevisiae*, including the well-studied Pdr5. Pdr5 (ScABCG1b/ YOR153W) plays roles in endocytosis-vacuolar degradation process, modulation of intracellular levels of steroid hormones, efflux of weakly charged organic compounds, anticancer drugs and various cellular detoxification processes that could be attributed to its highly diverse substrate specificity. Pdr10 (ScABCG1c/ YOR328W), homolog of Pdr5, acts as negative regulator of mechanisms incorporating Pdr12 in the membranes [122]. This group harbored *B*. *cinerea* BcatrD (BcABCG1b/ BC1G_05954), the mutants of which exhibited sensitivity towards sterol demethylation inhibitor (DMI) fungicides like oxpoconazole [123]. Another homolog, MgAtr7 (MgABCG1) interestingly was involved in iron homeostasis [124]. The expression of *MgAtr7* serially decreased during plant infection phases- spore germination (1 dpi), slow symptomless invasive growth (4 dpi), rapid invasive growth (9 dpi), necrotrophic-sporulation onset (14 dpi) and extreme necrotrophic (21 dpi) stages during interaction with wheat leaves compared to the mycelia from log-phase cultures [45]. This behavior of *MgAtr7* is contrasting to *Abc1*-like homolog, i.e. MgABCG2, harbored by its neighboring clade, where MgABCG2 was noted with higher gene expression across 1 dpi to 14 dpi in the same experiment (S5 Table) [45]. Hence it could be suggested that there might be different regulatory mechanisms for these two ABC-G members. It is likely that ABC-G Group I members might be indirectly aiding virulence through detoxification or efflux of organic and inorganic compounds. Group I is a small group dominated by ABC-G from the Model species and could help link annotations of characterized ABC-Gs to the Dothideomycetes ABC-Gs such as LmABCG1 and MgABCG1 (S10 Table).

Group II: This group possessed Abc1 (MoABCG2/MGG_13624) of *M*. *oryzae*, which is a pathogenic factor in rice blast infection, as revealed by an insertional mutagenesis screen [125]. One to two *Abc1*-like genes were present in all the fungal genomes analyzed us, but were absent in *B*. *dothidea*, *C*. *fulvum* and *S*. *cerevisiae*. *Abc1* was up-regulated during appressoria development on hydrophobic surface *in vitro* [61] and during compatible and incompatible interactions with rice leaves in spore germination-epidermal penetration phase (24 hpi) [62]. However, it had significantly lower expression levels during invasive growth stage (3 dpi) in compatible interactions with barley [34] (S2–S6 Tables). Another Group II member, *FgABC3* (FgABCG2b/ FGSG_04580) was functionally characterized using mutant based studies and was shown to confer tolerance against fungicides like triazoles and fenarimol [18]. This gene was expressed at higher levels during fungal mycelia development on wheat compared to mycelia *in vitro* [81]. This group also harbored *M*. *graminicola* MgABCG2, which was significantly and serially expressed during *in planta* spore germination (1 dpi), slow symptomless invasive growth (4 dpi), rapid invasive growth (9 dpi) and necrotrophic onset (14 dpi) stages during interaction with wheat leaves compared to mycelia from log-phase cultures [45]. This emphasizes the importance of this fungal gene in plant infection stages and thus it could be further functionally characterized as one of the potential factors that drives the fungal invasion over the plant host. Phylogenetic arrangement of this group showed clear division between subclades of Dothideomycetes and the other ABC-G members. As an exception, MphABCG2a was placed within non-Dothideomycetes subclade.

Group III: This group featured up to three ABC-G proteins (*C*. *higginsianum*) in the analyzed fungal genomes; however, they were absent in as many as eight fungal species including *S*. *cerevisiae* (Fig 10). This group was dominated by ABC-G members from the Model fungi, as only eight out of 14 Dothideomycetes members were represented here. During our survey, MoABCG3b (MGG_07848) showed up-regulated gene expression in five different experimental conditions- *in vitro* appressorial development on plant surface mimicking hydrophobic plates compared to mycelia *in vitro* [61]; spore germination-epidermal penetration phase (24 hpi) during *in planta* compatible and incompatible interactions with rice leaves compared to germinated spores *in vitro* [62]; and invasive growth stage (3 dpi) while in compatible interactions with both rice and barley compared to mycelia *in vitro* [34] (S5 and S6 Tables). *Abc1* (Group II) was co-expressed along with its paralog MoABCG3b from Group III with similar expression pattern across different infection stages, suggesting that they could be regulated or functioning together during these infection stages. A paralog of MoABCG3b, MoABCG3a (MGG_07375) was highly co-expressed during invasive growth stage (3 dpi) in compatible interactions with both rice and barley (S6 Table) [34]. This group also possessed ChiABCG3c (CH063_13804) whose expression serially decreased from appressorial pre-penetration (22 hpi) to early biotrophic stage (40 hpi) towards necrotrophic phase (60 hpi) (S5 Table) [43]. Such down-regulated genes raise questions whether the fungus is selecting alternative transporters with similar functions or if their regulation is targeted by any plant defense compound or they are specific to plant defense molecules produced in the earlier phases of infection. The Dothideomycetes BdoABCG3, which was closer to the well annotated ABC-Gs, can be evaluated for functional roles through experiments similar to those in which their neighboring annotated ABC-G were implicated.

Group IV: This group possessed the well-known virulence factor of *M*. *graminicola*, MgAtr4 (MgABCG4), which has been shown to play a major role in fungal colonization in the substomatal cavities of wheat leaves and aids in intercellular fungal growth in the leaf apoplast [17]. Our gene expression data compilation showed that it had low transcript levels during the rapid invasive growth phase (9 dpi) in interaction with wheat leaves compared to mycelia from log-phase cultures (S3 and S5 Tables) [45]. In *C*. *higginsianum*, the expression of ChiABCG4 (CH063_00247) was highest during biotrophic stage (S5 Table) (40 hpi) compared to the necrotrophic stages (60 hpi) of infection on *Arabidopsis* [43]. Overall, these examples suggested that these genes might be important during early stages of plant infection than later stages. Almost all the fungi considered in the present study (16), were represented in this group with a single gene, except *F*. *graminearum*, which showed two, *B*. *dothidea* with three and *M*. *phaseolina* with five genes; while *M*. *oryzae* and *S*. *cerevisiae* possessed none. Phylogenetic analysis revealed separation of ABC-G from Dothideomycetes and Model fungi into several small clades. Functional or gene expression annotations were seen on both sides broadening the scope for associating possible functions (S10 Table).

Group V: This is a small phylogenetic group of ABC-G proteins with five proteins from *S*. *cerevisiae*, three from *B*. *dothidea* and one protein from *M*. *phaseolina*. The representatives of this group, *S*. *cerevisiae* Aus1 (ScABCG5b/ YOR011W) and Pdr11 (ScABCG5a/ YIL013C) carry out sterol import [126]. Snq2 (ScABCG5c/ YDR011W) of this group also confers cation resistance [127], mediates sterol transport [128], can efflux caffeine [129] along with imparting resistance to photosensitizers like cercosporin [130]. Two additional *S*. *cerevisiae* ABC-G transporters, Pdr18 (ScABCG5d/ YNR070W) and Pdr12 (ScABCG5e/ YPL058C), were present in this group. Pdr18 is known to increase yeast ethanol tolerance and significantly aids in higher bio-ethanol production [131]. It also controls plasma membrane sterol composition along with its various multi-drug resistance roles [132]. Pdr12 is a known organic acid transporter [133]. The Dothideomycetes members BdoABCG5a/5b/5c and MphABCG5 were closer to these *S*. *cerevisiae* ABC-G proteins and could be experimentally validated for such putative functions.

Group VI: BcatrA (BcABCG6a/ BC1G_03332) was the representative member of Group VI. Disruption of *BcatrA* gene in *B*. *cinerea* rendered sensitivity to cycloheximide and catechol in a heterologous (yeast) ABC transporter mutant system. Its involvement during initial stages of pathogenesis has also been reported [134]. BcatrA-like VdaABCG6 (VDAG_08191) was also up-regulated significantly during microsclerotia formation compared to the conidial stage [69]. In our phylogenetic analysis, the BcatrA-like Group VI members were closely clustered with YOL075C-like (Group VII) members but distantly from BcatrB-like (Group XI) members. ABC-G members of Group VI were absent in *M*. *oryzae* and *S*. *cerevisiae*. All the other fungi that we studied were represented in this clade with either one or two of such genes. The phylogenetic analysis revealed distinct division between ABC-G from Dothideomycetes and Model fungi. However, Dothideomycetes ABC-G proteins like MgABCG6, MfABCG6b, CfABCG6 and DsABCG6 could be more eligible for associating functions of closely clustered characterized ABC-Gs (S10 Table).

Group VII: The *S*. *cerevisiae* YOL075C (ScABCG7) from this group has not been characterized in detail. However, we mined the public data and found interesting information regarding the members of this group. MoABCG7 (MGG_10410) was highly expressed during appressorial development on plant surface mimicking hydrophobic surface [61] and epidermal penetration stage (1 dpi) during interactions with rice leaves [62], while it was down-regulated significantly across invasive growth phase (3 dpi) in compatible interactions with barley compared to mycelia *in vitro* [34] (S5 and S6 Tables). This suggests that Group VII members could play a role during early infection stages, while exhibiting suppressed function across later stages. A single gene each of Group VII was present in 14 of the fungi analyzed in this study, except *B*. *cinerea*, *C*. *fulvum*, *D*. *septosporum*, *P*. *tritici-repentis* and *V*. *inaequalis* and the *Mycosphaerella* fungi that possessed none. The phylogenetic clades of this group showed clear distinction between Dothideomycetes and ABC-G proteins from the Model fungi, while ScABCG7 formed outgroup.

Group VIII: This group did not possess any well-characterized ABC member. However, our survey of previous publicly available data showed five members from this group with interesting gene expression information. In an experiment studying the infection cycle of *M*. *graminicola* on wheat, MgABCG8b (XP_003853355.1) was significantly up-regulated during spore germination stage (1 dpi), while MgABCG8a was down-regulated during extreme necrotrophic stage (21 dpi) of infection cycle compared to mycelia from log-phase cultures [45]. Other examples of ABC-G genes with low gene expression levels during biotrophic-necrotrophic transition, extreme necrotrophic and invasive growth stages of infection cycle of different fungi in multiple experiments (S5 Table) were also found and are highlighted in Fig 10, Group VIII. All the considered fungi in this study, except *S*. *cerevisiae*, were represented in Group VIII with one or two genes. ABC-G members of this group formed separate clades of Dothideomycetes and ABC-G members from the Model species, except BcABCG8 and VdaABCG8 that clustered with the Dothideomycetes members. This helped us list the potential Dothideomycetes candidates from this group for further characterization (S10 Table).

Group IX: Abc2 (MoABCG9/MGG_00447) represented this group, whose gene disruption in *M*. *oryzae* identified it as sensitivity determinant of sterol demethylase inhibitor fungicides bitertanol, myclobutanil and tebuconazole (S4 Table) [135]. Additionally, *Abc2* prominently enhanced its expression during appressorial differentiation (up to 16 h), invasive growth stage (3 dpi) in compatible interactions with rice compared to mycelia *in vitro* and during epidermal penetration phase (1 dpi) of compatible and incompatible interactions with rice compared to *in vitro* germinated spores [34,61,62] (S5 and S6 Tables). Further, *B*. *cinerea* Bmr1 (BcABCG9/ BC1G_00425) conferred resistance against fungicides like polyoxin and iprobenfos (S4 Table) [136]. This suggested that the members of this group may be active participants in fungicide tolerance activities. A single ABC-G transporter of this group was present in each of the analyzed fungi, except in *S*. *cerevisiae*. The phylogenetic tree revealed clear distinctions between Dothideomycetes and non-Dothideomycetes ABC-G subclades, wherein MgABCG9b as an exception was more closely clustered with ABC-G proteins from Model fungi. This makes MgABCG9b a promising candidate for evaluating functions similar to BcABCG5 (Bmr1).

Group X: The *S*. *cerevisiae* Adp1 (ScABCG10), the representative of Group X, is a potential glutathione exporter [137]. Although, not much is known about Adp1 and its homologs, we were able to trace the information about them that can be added to the annotation of this group. *Adp1*-like FgABCG10b (FGSG_05076) was up-regulated when the fungal mycelia was treated with Tebuconazole [44], while *M*. *oryza*e MoABCG10 (MGG_01563) was down-regulated during invasive growth stage (3 dpi) in compatible interactions with barley compared to *in vitro* mycelia [34]. The *M*. *graminicola* MgABCG10a also showed six-folds increase in gene expression during rapid invasive growth (9 dpi) and four-folds increase in necrotrophic phase (14 dpi) of wheat infection compared to mycelia from log-phase cultures [45] (S5 and S6 Tables). All the analyzed fungi possessed Group X genes ranging from one to three, except *C*. *higginsianum* that possessed none. The phylogenetic clade of this group lacked clear distinction between the Model and Dothideomycetes members and hence further studies on at least a few members of this group could help target analyses on the others.

Group XI: This group was represented by BcatrB (BcABCG11a /BC1G_04420) which has been identified as a virulence factor in several experiments (S3 and S4 Tables). *B*. *cinerea* exhibited reduced virulence when *BcatrB* gene replacement-mutants were inoculated on tomato leaves [123]. It also acted as a virulence factor against *A*. *thaliana*, in addition to conferring resistance towards camalexin, a phytoalexin secreted by this host plant [138]. In addition, *BcatrB* gene replacement rendered the mutant sensitive towards the fungicide fenpiclonil and the phytoalexin resveratrol secreted by its host grapevine [139]. Further, *BcatrB* also helped in providing tolerance against iprodione, cyprodinil and phenylpyrrole-based fludioxonil fungicides [140]. A study on the homolog of *BcatrB*, *MgAtr5* (MgABCG11), showed that *MgAtr5* deletion mutants of *M*. *graminicola* responded with increased sensitivity towards the putative wheat defense compound resorcinol and the grape phytoalexin resveratrol. In addition, *MgAtr5* also contributed to the tolerance for plant metabolites like- berberine and camptothecin [141].

The expression of another homolog FgABCG11b (FGSG_08830) was highly up-regulated when *F*. *graminearum* mycelia were treated with the fungicide Tebuconazole [44]. In different experiments, MoABCG11 (MGG_10277) of *M*. *oryzae* had significantly enhanced gene expression during appressorial development on plant surface mimicking hydrophobic coats [61] and across invasive growth stage (3 dpi) in compatible interactions with rice, compared to mycelia *in vitro* [34]. Few cases of *Colletotrichum* fungi were reported where their ABC-G transporters belonging to Group XI showed modulation in their gene expression during different stages of fungal invasion on *Arabidopsis* [43] (S5 and S6 Tables). All these examples suggested that Group XI members possess high substrate diversity, which could provide it the ability to act as a virulence factor and/or protect against various kinds of anti-fungal molecules. Group XI members were contributed by all the analyzed fungi with one gene each, except *S*. *cerevisiae* that possessed none. The phylogenetic clade of this group was divided into two distinct sub-groups; however, no clear separations between Dothideomycetes and the Model fungi were seen. Dothideomycetes ABC-G genes like MfABCG11 and DsABCG11 were closely clustered with the functionally annotated MgABCG11 and BcABCG11a. Hence, these Dothideomycetes ABC-G can be evaluated for similar functions in similar experimental conditions.

#### Subfamily I

Subfamily I includes two types of ABC proteins- Caf16-like and Ydr061w-like and both types are soluble proteins lacking TMD [10]. In plants, bacterial-type ABC-I have been implicated in phosphatidic acid transport into the plastids as well as in aluminum tolerance [56]. In fungi, Caf16 (YFL028C) of *S*. *cerevisiae* is a subunit of CCR4-NOT transcriptional regulatory complex associated with regulating mRNA initiation, elongation and mRNA degradation [142]. In spite of being an important transcriptional regulatory factor, Caf16 still remains poorly studied. In the ABC-I proteins we analyzed, we noted that Caf16-like proteins contained a single NBD and Ydr061w-like members possessed two (S8 Table). In our study, ABC-I phylogenetic tree displayed two distinct groups corresponding to Caf16-like Group I and Ydr061w-like Group II (Fig 11), similar to the two groups identified by Kovalchuk and Driessen [10]. Both the groups showed separation between subclades of Dothideomycetes and non-Dothideomycetes members. Caf16-like proteins were present in all analyzed fungi, except in *S*. *nodorum*. Similarly, Ydr061w-like proteins were possessed by all, except *V*. *dahliae* (S8 Table). It was surprising to note that the ABC-I members were absent from these Dothideomycetes, as only some of the primitive fungal species such as *Encephalitozoon cuniculi* and *Batrachochytrium dendrobatidis* lacked them as reported earlier ([10] and references therein).

We could trace a Caf16-like ABC protein, MgABCI1 in *M*. *graminicola*, to an RNAseq experiment [45], which revealed down-regulation of this ABC-I gene during *in planta* (wheat) conditions at spore germination (1 dpi), slow symptomless invasive growth (4 dpi) and onset of rapid invasive growth (9 dpi) stages compared to mycelia from log-phase cultures (S5 Table). In contrast, among the Ydr061w homologs, MgABCI1 gene expression was down-regulated *in planta* in rice compared to mycelia growing *in vitro* (S6 Table). We strongly suggest that targeted investigations need to be carried out on Caf16-like and Ydr061w-like members to identify their functions and evaluate whether their expressions are modulated during plant-pathogenic interactions.

## Conclusions

In this study, we exploited the genome sequence data of 21 fungi and identified, classified, characterized and compared the repertoire of ABC proteins of Dothideomycetes phytopathogens and other selected Ascomycetes species. Our analysis revealed phylogenetic relationships among members of the eight major ABC protein subfamilies. Our efforts to perform a systematic review of gene expression and functional data on homologous fungal ABC superfamily members and overlay of the reviewed information on the phylogenetic trees of individual subfamilies provided us with an array of Dothideomycetes ABC proteins that might have a role in plant-pathogen interactions and/or response to antifungal chemicals, which could be forwarded further for experimental testing. In all, we listed 86 Dothideomycetes ABC proteins that we propose as top candidates for experimental validation (S10 Table). This would help in facilitating targeted research on ABC proteins and their roles in pathogenicity and fungicide tolerance.

## Supporting information

S1 Table Analyzed fungal species, their taxonomic classification and genome sequence references. (XLSX)

S2 Table Reference ABC protein sequences and their details. (XLSX)

S3 Table Functional information on ABC genes retrieved through PHI-Base. (XLSX)

S4 Table Functional information on ABC genes retrieved through literature sources. (XLSX)

S5 Table Differentially expressed ABC genes in various experimental conditions compiled and curated from literature sources. (XLSX)

S6 Table Differentially expressed ABC genes identified by re-analyzing microarray datasets obtained from Mathioni *et al*. (2011). (XLSX)

S7 Table Repertoire and classification of ABC proteins identified from the fungal species analyzed in the present study. (XLSX)

S8 Table Identified ABC proteins, their genomic co-ordinates, sequences, alignment and domain analysis results and other related information. (XLSX)

S9 Table Correlation between number of ABC genes, genome size and total genes. (XLSX)

S10 Table List of top Dothideomycetes ABC protein candidates recommended for experimental validation of their roles in host-pathogen interactions and/or fungicide tolerance; based on gene expression or functional analysis data on the Ascomycetes homologs and phylogenetic analysis results of the present study. (XLSX)

S1 File Newick file for the phylogenetic tree represented in Fig 2 (Fungal species). (NWK)

S2 File Newick file for the phylogenetic tree represented in Fig 3 ("ABC-Dot"). (NWK)

S3 File Newick file for the phylogenetic tree represented in Fig 4 (ABC-A). (NWK)

S4 File Newick file for the phylogenetic tree represented in Fig 5 (ABC-BFT). (NWK)

S5 File Newick file for the phylogenetic tree represented in Fig 6 (ABC-BHT). (NWK)

S6 File Newick file for the phylogenetic tree represented in Fig 7 (ABC-C). (NWK)

S7 File Newick file for the phylogenetic tree represented in Fig 8 (ABC-D). (NWK)

S8 File Newick file for the phylogenetic tree represented in Fig 9 (ABC-E and F). (NWK)

S9 File Newick file for the phylogenetic tree represented in Fig 10 (ABC-G). (NWK)

S10 File Newick file for the phylogenetic tree represented in Fig 11 (ABC-I). (NWK)
